# A comparison of RNA-seq and exon arrays for whole genome transcription profiling of the L5 spinal nerve transection model of neuropathic pain in the rat

**DOI:** 10.1186/1744-8069-10-7

**Published:** 2014-01-28

**Authors:** James R Perkins, Ana Antunes-Martins, Margarita Calvo, John Grist, Werner Rust, Ramona Schmid, Tobias Hildebrandt, Matthias Kohl, Christine Orengo, Stephen B McMahon, David LH Bennett

**Affiliations:** 1 Department of Structural and Molecular Biology, University College London, Darwin Building, Gower Street, London WC1E 6BT, UK; 2 Laboratorio de Investigacion, Fundacion IMABIS, Avda. Jorge Luis Borges nº15 Bl.3 Pl.3, 29010, Malaga, Spain; 3 The Wolfson Centre for Age-Related Diseases, Wolfson Wing, Hodgkin Building, King’s College London, Guy's Campus, London Bridge, London SE1 1UL, UK; 4 Boehringer Ingelheim Pharma GmbH & Co. KG, Target Discovery Research Germany, Birkendorferstraße 67, 88397, Biberach an der Riß, Germany; 5 Department of Medical and Life Sciences, Furtwangen University, Jakob-Kienzle-Str. 17, D-78054 VS-Schwenningen, Germany; 6 Nuffield Department of Clinical Neurosciences, Level 6, West Wing, John Radcliffe Hospital, Oxford OX3 9DU, England

**Keywords:** Whole-genome transcription profiling, Exon arrays, Microarrays, RNA-Sequencing, RNA-seq, Next generation sequencing, Spinal nerve transection, Nerve injury, Neuropathic pain, Differential gene expression

## Abstract

**Background:**

The past decade has seen an abundance of transcriptional profiling studies of preclinical models of persistent pain, predominantly employing microarray technology. In this study we directly compare exon microarrays to RNA-seq and investigate the ability of both platforms to detect differentially expressed genes following nerve injury using the L5 spinal nerve transection model of neuropathic pain. We also investigate the effects of increasing RNA-seq sequencing depth. Finally we take advantage of the “agnostic” approach of RNA-seq to discover areas of expression outside of annotated exons that show marked changes in expression following nerve injury.

**Results:**

RNA-seq and microarrays largely agree in terms of the genes called as differentially expressed. However, RNA-seq is able to interrogate a much larger proportion of the genome. It can also detect a greater number of differentially expressed genes than microarrays, across a wider range of fold changes and is able to assign a larger range of expression values to the genes it measures. The number of differentially expressed genes detected increases with sequencing depth. RNA-seq also allows the discovery of a number of genes displaying unusual and interesting patterns of non-exonic expression following nerve injury, an effect that cannot be detected using microarrays.

**Conclusion:**

We recommend the use of RNA-seq for future high-throughput transcriptomic experiments in pain studies. RNA-seq allowed the identification of a larger number of putative candidate pain genes than microarrays and can also detect a wider range of expression values in a neuropathic pain model. In addition, RNA-seq can interrogate the whole genome regardless of prior annotations, being able to detect transcription from areas of the genome not currently annotated as exons. Some of these areas are differentially expressed following nerve injury, and may represent novel genes or isoforms. We also recommend the use of a high sequencing depth in order to detect differential expression for genes with low levels of expression.

## Background

Gene expression studies can be used to provide insights into the molecular mechanisms underlying the onset and maintenance of pain [1-5]. Such approaches can be hypothesis-driven, assessing the expression of preselected candidate molecules, or hypothesis-independent, interrogating gene expression at the genome-wide level.

Microarrays have been used extensively to investigate transcriptional changes that occur in different parts of the central and peripheral nervous systems [4]. Such studies have led to the discovery of novel pain-related genes, such as the Potassium voltage-gated channel subfamily S member 1, KCNS1 [2], GTP cyclohydrolase 1, GCH1 [6] and the neuropeptide VGF nerve growth factor inducible [7]. In recent years RNA-sequencing (RNA-seq) has emerged as an alternative platform for high-throughput transcriptional profiling [8]. The platform has been used in a variety of studies, but so far only one report has described the use of RNA-seq to measure gene expression changes in the peripheral nervous system in an experimental model of pain [9]. It has also been used to perform microRNA profiling following sciatic nerve injury [10] and to study gene expression changes in the pre-frontal cortex following spared nerve injury [11].

Microarray chips measure the expression of thousands of genes in a sample by quantifying the hybridisation of fragmented cDNA derived from gene-transcripts to a set of complementary probes specifically designed to detect a set of genes or transcripts. They have been used to study a variety of biological systems [12]. However, the use of probes leads to a number of drawbacks, including non-specific binding and signal saturation [13], which can negatively affect the measurement of expression for both lowly and highly expressed genes. Furthermore, microarray design is based on prior knowledge of the transcriptome and therefore microarrays can only interrogate a subset of known (or predicted) transcripts.

RNA-seq represents an alternative to microarrays [8]. It uses high throughput sequencing technology to investigate RNA expression [14] and allows the quantification of thousands of transcripts within a cell line or tissue without the need for *a priori* knowledge of the transcriptome. This “agnostic” approach represents a major advantage over microarrays, allowing the discovery of new transcript variants, novel genes and the annotation of less well characterized genomes [15,16].

RNA-seq technology also suffers from drawbacks. The output of an RNA-seq experiment consists of millions of reads, short sequences of cDNA derived from RNA molecules. These reads must be mapped to a reference genome in order to identify the genomic location of the originating transcript and thus quantify expression [17]. Thus, analysis can be computationally expensive and time consuming. A common challenge arises due to the presence of reads that cannot be mapped to the genome. This can be due to genomic differences (such as polymorphisms) between the sample and the reference genome or erroneous base calling by the sequencing technology [17]. Conversely, RNA-seq reads may map to more than one genomic location. Such ambiguous reads can lead to imprecise gene quantification.

An important consideration when designing an RNA-seq experiment is sequencing depth, the number of reads generated per sample. In general it is expected that the higher the sequencing depth the more accurately the transcriptome of the tissue of interest is quantified [18,19]. This is particularly important for the accurate detection of lowly expressed genes, where problems related to sampling error can lead to an over or under-estimation of transcript abundance. However, the cost of the experiment will also increase with sampling depth.

In addition to the technical pros and cons of both platforms, price is an important factor. Although RNA-seq costs are decreasing, microarrays remain more affordable, and data analysis is more standardised and easily implemented [16]. RNA-seq, on the other hand, demands more computational power and bioinformatics expertise; therefore it is important to determine to what extent the additional knowledge generated by RNA-seq experiments outweighs the computational demands and economic costs.

A large number of comparisons between RNA-seq and gene expression microarrays have been reported, covering a wide variety of different experimental designs, platforms, organisms, tissues, cell lines and experimental interventions. Table 1 shows an overview of these previous studies. So far, no direct comparison has been made using exon arrays for non-human, heterogeneous tissue from different individuals.

**Table 1:** Previous studies comparing microarray and sequencing platforms for the measurement of gene expression

**Study**	**Array platform**	**Sequencing platform**	**Species**	**Tissue/cell line**	**Replication**	**Experimental intervention/design**
Marioni et al. 2008 [20]	Affymetrix HG-U133 Plus 2.0	Illumina Genome Analyzer	Human	Liver/kidney	Technical replications (3 per tissue for microarray, 7 different flow cell lanes for RNA-seq).	All RNA was taken from a single human male. Aliquots from each sample were then used for RNA-sequencing and microarray analysis.
Bradford et al. 2010 [21]	Affymetrix Human Exon 1.0 ST	Applied Biosystems SOLiD v3 platform	Human	MCF-7 and MCF-10a breast cancer lines	Technical replication (2 x MCF-7, 1 x MCF-10). Samples hybridised in triplicate to microarrays.	RNA analysed on the SOLiD platform and the same RNA samples hybridised in triplicate to Affymetrix Exon 1.0ST arrays.
Bottomly et al. 2011 [22]	Affymetrix MOE 430 2.0 and Illumina MouseRef-8 v2.0	Illumina GA IIx	Mouse	Striatum	Biological replication, independent groups used for different technologies.	B6 strain mice were compared to D2 strain. For RNA-seq, 10 B6 and 11 D2 were used; for Affymetrix arrays 7 D2, 10 B6; for Illumina arrays 12 D2 12 B6. A subset of this group of mice were also used for RNA-seq.
Toung et al. 2011 [23]	Affymetrix HG Focus Array	Illumina 1G Genome Analyzer	Human	B-cells	Biological replicates (20 unrelated individuals). Independent samples (from same individuals) were used for different technologies.	B-cell lines were taken for 20 different individuals (10 male, 10 female). Cells were grown and total RNA extracted.
Su et al. 2011 [24]	Affymetrix Rat Genome 230 2.0	Illumina GA II	Rat	Kidney	Biological replication (4 rats per condition).	Eight rats in total, 4 were administered with aristolochic acid, 4 with control vehicle. RNA was extracted from kidneys of each rat; each RNA sample was assayed using RNA-seq and miroarrays
Fu et al. 2009 [25]	Affymetrix Human Exon 1.0 ST	Illumina Solexa Sequencer (precise model name not given)	Human	Brain	Biological replication (two groups of 5 pooled individuals).	Two independent samples were used, each containing pooled mRNA from 5 adult human individuals. These samples were used as input for RNA-seq, microarray and proteomic analysis.
Griffith et al. 2010 [26]	Affymetrix Human Exon 1.0 ST and Nimblegen custom array	Illumina GA II	Human	Colorectal cancer cell-lines	One sample per condition.	5-fluorouracil resistant cell lines compared to non-resistant lines. The same input was used for microarrays and RNA-seq.
Bullard et al. 2010 [27]	Affymetrix U133 Plus 2.0	Illumina GA II	Human	Brain reference DNA and universal human reference DNA	Technical replication.	Various experimental designs were employed in order to teaste apart the effects of flow cell and library preparation on the results.
Kogenaru et al. 2012 [28]	Agilent custom array	Illumina GA IIx	*Xanthomonas citri subsp* ***.*** *citri* ***.***	Whole organism	Biological replication (3 replicates per strain).	Comparison was made between wild-type and hrpX mutant strains. Biological replicates of each strain were grown in culture and the RNA was extracted.
Sîrbu et al. 2012 [29]	Affymetrix and dual-channel microarrays	Illumina GA II	Drosophila	Embryo development (time-series)	Technical replicates were used for RNA-seq, biological replicates were used for microarray.	Datasets were analysed and compared in terms of “reference” genes, which were highly likely to be expressed during embryogenesis. Several other technical measurements were also taken, including clustering and differential expression measurements.
Sekhon et al. 2013 [30]	NimbleGen custom array	Illumina GA II	Maize	18 selected tissues representing 5 organs	Biological replicates, compared to historical dataset.	Samples were assayed by both technologies, and compared in terms of expressed genes and correlation.
Mooney et al. 2013 [31]	Affymetrix Canine Genome 2.0	Illumina Hiseq 2000	Dog	B-cell lymphoma	Biological replication; same samples used for both technologies (10 case, 4 control samples).	Investigation into the difference between technologies in terms of technical biases and pathways found.
Malone and Oliver 2011 [32]	Nimblegen custom array	Ilumina GA I	*Drosophila pseudoobscura*	Head	Biological replicates (four for microarray; one of these replicates used for RNA-seq).	RNA from males was compared to RNA from females. Four distinct RNA libraries were produced, with each library produced using 500–600 individual fly heads.

In this study we investigate whether RNA-seq offers an advantage over microarrays for the study of differential gene expression within dorsal root ganglia (DRG) following nerve injury using the L5 spinal nerve transection (SNT) model of neuropathic pain in the rat [33]. Expression changes induced by this injury have been well characterised by microarrays [1,4,34], proteomics [35] and reverse transcription quantitative PCR (RT-qPCR) [1]. We have used technical replicates of the same biological samples and subjected them to exon expression array and RNA-seq analysis. This circumvents confounding effects brought about by comparing distinct technologies using historical data from previous/independent studies. We demonstrate the technical superiority of RNA-seq over microarrays in terms of sensitivity (ability to measure transcripts) and ability to detect differential gene expression. The latter is particularly important in the context of pain and nerve injury, as RNA-seq detects a large number of highly dysregulated genes, which may represent novel candidate pain genes. We also investigate the effects of increased sequencing depth on the results of an RNA-seq experiment, and the ability of RNA-seq to detect expression originating from unannotated genomic regions.

## Results

Spinal nerve transection (SNT) is a widely used experimental model of neuropathic pain, associated with profound changes in gene expression in dorsal root ganglia (DRG) [1]. It therefore represents an excellent model for assessing the relative merits of RNA-seq in comparison with microarrays in the context of pain. DRG tissue was harvested at day 7 post transection, a time point at which altered gene expression and associated pain-related behaviour are well established [36].

**Figure 1:**
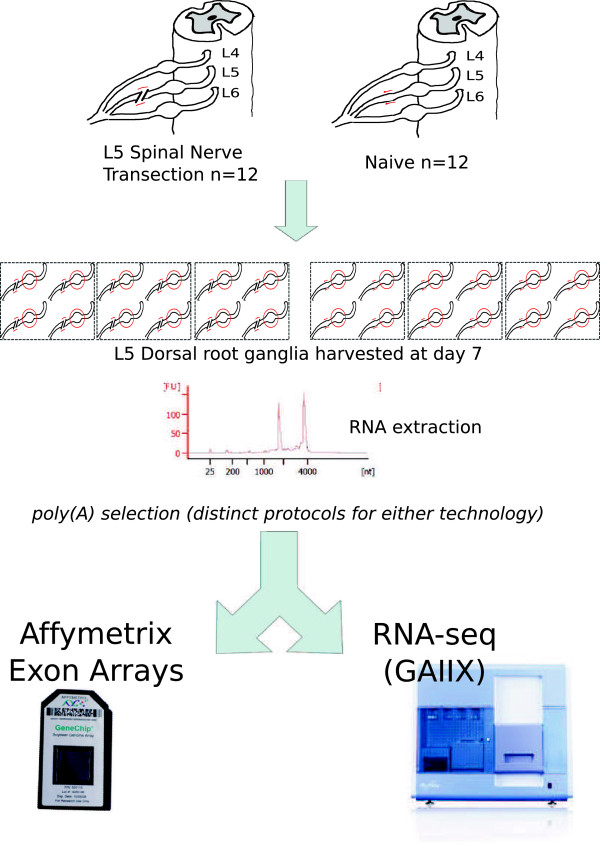
**Experimental outline.** L5-spinal nerve transection (SNT) was performed on twelve male Wistar rats. L5 DRG tissue was harvested 7 days after surgery and tissue from 4 animals was pooled for RNA extraction; naive tissue was used as control. Total RNA samples (n = 3 SNT, n = 3 naive) were divided into technical replicates used in parallel for microarray analysis (Affymetrix Rat Exon 1.0 ST arrays) and RNA-seq in the Illumina GAIIx platform, in both cases following poly(A) enrichment.

We have performed RNA-seq and microarray analysis in parallel on technical replicates of the same biological samples. This design allowed us to avoid confounding effects due to biological variability and other differences in sample preparation. Poly(A) enriched RNA derived from the L5 DRG 7 days following L5-SNT and from naive L5 DRG tissue was subjected to microarray analysis and RNA-seq, as outlined in Figure 1. Therefore, in total there were three biological replicates per condition; each of these six replicates was subdivided to produce technical replicates, one of which was used for microarray analysis, the other for RNA-seq. The aims of our experiment were three-fold: Firstly, to compare the ability of both methods to measure gene expression and identify differentially expressed (DE) genes. Secondly, to compare three distinct read depths for RNA-seq in order to investigate its impact on the detection of transcriptional changes across different levels of gene expression. Thirdly, to investigate changes in gene expression influenced by expression from areas outside of annotated exons (areas within annotated 5’ and 3’ ends of genes that are not annotated as exons in the reference genome) within the DRG following SNT.

### Mapping sequenced reads to the genome

**Figure 2:**
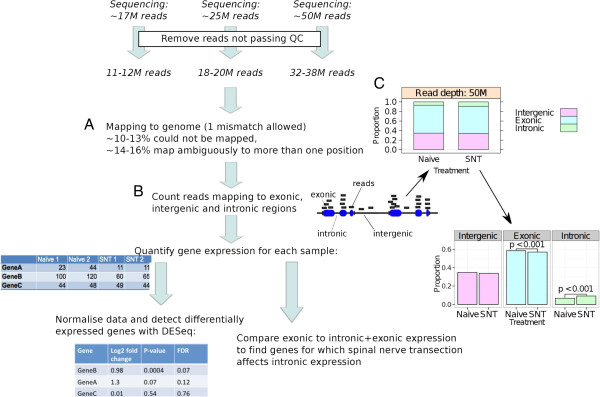
**RNA-seq procedure and RNA-seq analysis pipeline.** cDNA libraries were produced for each sample from poly(A) enriched RNA. These were sequenced to three distinct read depths (~17, ~25 and ~50 M reads/sample). Reads not passing the Illumina quality filter were discarded. **A)** Filtered reads were mapped to the genome, allowing a maximum of one mismatch between the sequence and the reference genome (Rn5); reads that could not be mapped to the reference genome or that could be mapped to more than one genomic location (ambiguous reads) were discarded. **B)** The remaining reads were mapped onto the genome and classified as exonic, intronic or intergenic as described in the Methods section. **C)** Stacked bar charts, showing the proportions of exonic, intronic and intergenic reads, at a 50 M read depth (the same pattern was obtained at 17 M and 25 M read depths). The unstacked barcharts show there is a significantly higher proportion of reads that align to intronic regions in SNT samples than in naive samples, and that the proportion of reads mapping to exonic regions is significantly higher in naive samples than in SNT samples. P-values were calculated using the overdispersed logistic regression test described in the Methods section. Evidence of a difference between SNT and naive was found for intergenic reads, however this did not retain significance following the Bonferroni correction for multiple testing. Following alignment, gene expression was quantified by counting the number of reads mapping to each gene. Read counts were normalised by, and differential gene expression analysis was performed using DESeq. The effect of reads mapping to intronic regions on differential gene expression was assessed by comparing exonic expression to exonic and intronic expression.

We performed RNA-seq using the Illumina GAIIx platform. The protocol employed yielded 34 base-pair long reads, at three different sequencing depths per sample (~17, ~ 25 and ~50 M). Reads failing Illumina quality control due to ambiguous base calling were filtered out and the remaining reads were subsequently mapped to the reference rat genome (Rn5), permitting up to one mismatch when aligning reads to the genome (Figure 2A). Filtered reads that did not map to the genome or that could be mapped to more than one genomic location (ambiguous reads) were removed from the analysis.

**Figure 3:**
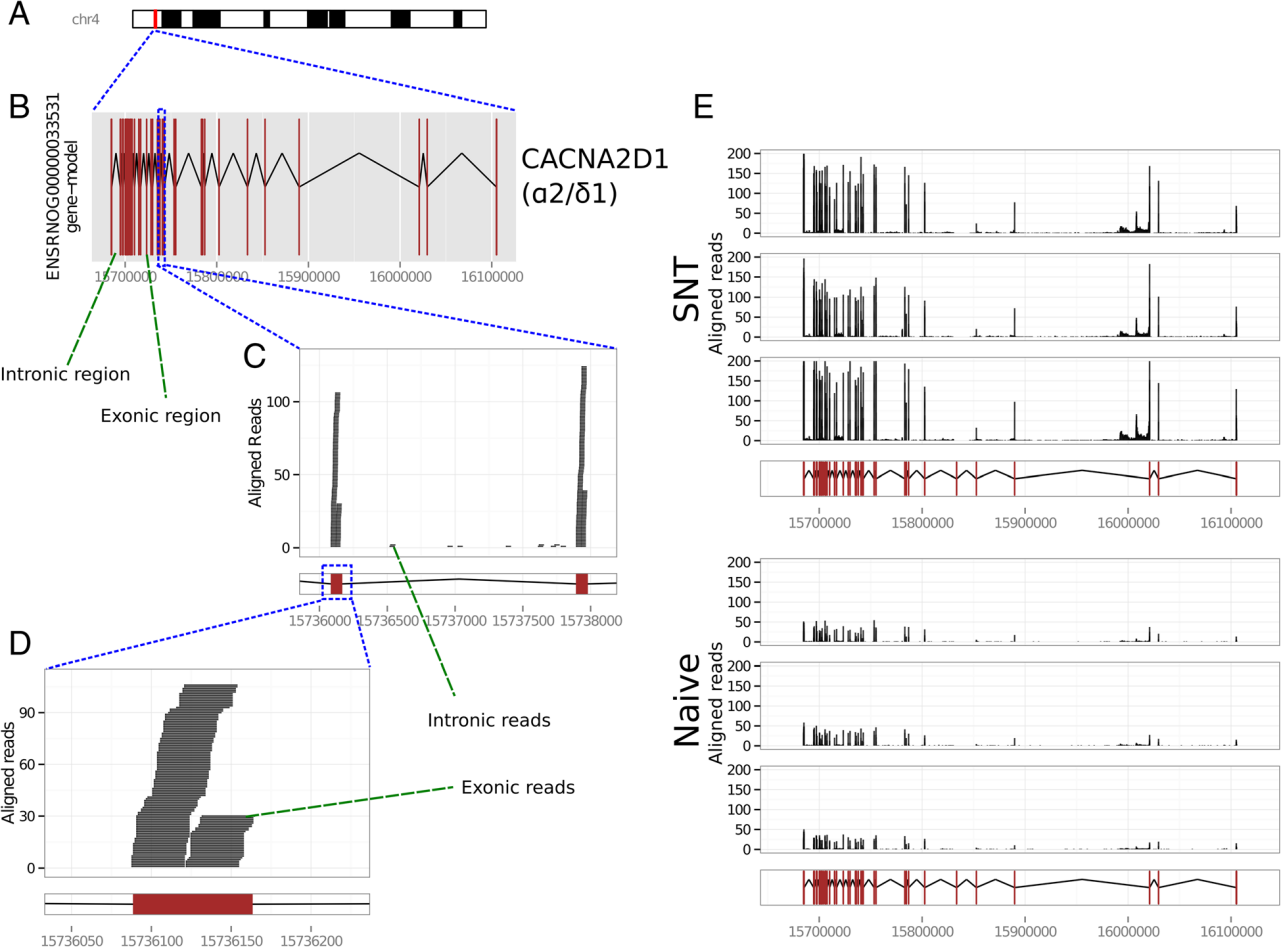
**Example of a genome graph for the Cacna2d1 gene (Calcium channel, voltage-dependent, alpha2/delta subunit 1). A)** The genomic location of the Cacna2d1 gene on chromosome 4, highlighted in red. **B)** The exonic/intronic structure of the gene; brown squares represent exons, the black links between them represent introns. **C)** A zoomed-in region of the gene, showing the positions where the sequenced reads align to the exons and introns of the gene (grey bricks), for a given RNA-seq sample (in this example, an SNT sample is shown, sequenced to a depth of 50 M reads). **D)** A further zoomed in region of the gene, showing the individual reads and the positions to which they align in greater detail. **E)** The genome graphs for all of the samples (shown for sequencing depth 50 M). Each row represents a different sample. When calculating fold change, the reads aligning to each exon are summed to produce the raw expression value for each sample, and then DESeq is used to compare these values between SNT and naive samples. Some reads align outside of known exons, this is explored further in the section “Intronic expression and its effect on fold change calculation”.

Mapped reads were categorised as exonic, intronic or intergenic as described in the Methods section (Figure 2B). An example of the read mapping procedure is giving in Figure 3, which shows the reads mapping to the genomic location of the gene Calcium Channel, Voltage-Dependent, Alpha 2/Delta Subunit 1 (Cacna2d1) for a given sample (Figure 3A-D) and for several samples (Figure 3E). The stacked bar charts (Figure 2C) illustrate the proportion of reads belonging to each category for naive and SNT samples, sequenced to a depth of 50 M reads. A high proportion of reads (~35%) map to intergenic regions, reflecting transcription from previously unannotated areas of the rat genome. In addition, when comparing the proportion of exonic and intronic reads across experimental groups, we observe a small but consistent and significant increase in the proportion of reads aligning to intronic regions following SNT (p < 0.001; see Methods section). These finding are suggestive of the increasingly recognised phenomenon of non-exonic, “dark matter” transcription [37-42]. At the level of individual genes, this observation may reflect an effect of SNT on alternative splicing that leads to inclusion of novel exons that have not yet been annotated, or the expression of unannotated overlapping/nested genes in the same genomic location. These putative nested genes may be protein coding or other polyadenylated RNA species. Detection of such an increase in non-exonic gene expression would not be possible using exon arrays, since they do not thoroughly profile the intronic regions of the genome (although for some genes there are probes that map to intronic regions). This increase in reads aligning to regions of genes that are not annotated as exonic was also found at read depths of 17 M and 25 M. In terms of intergenic aligning reads, there was a slight decrease following SNT, however this was not significant following Bonferroni correction for multiple testing.

### Proportions of the genome that can be measured by exon arrays and RNA-seq

RNA-seq data was aligned to the latest rat genome assembly, Rn5, whilst the microarray annotation available from NetAffx was designed for the previous version, Rn4. Rn5 contains annotation for 26405 Ensembl rat genes including protein-coding genes, miRNAs, ribosomal RNAs and pseudo-genes [43]. Rn4 contains annotation for 29516 rat genes. The intersection between these genome builds in terms of genes comprises 20914 Ensembl ids.

In order to compare gene expression between exon arrays and RNA-seq we first established the maximum number of genes that can theoretically be measured by each of the platforms, and the overlap between platforms. Microarrays contain a set of probes specifically designed to detect the expression of transcripts that have been previously characterised or computationally predicted. Affymetrix Rat Exon 1.0 ST arrays contain 17818 transcript-cluster mapping probesets, which have been assigned a range of confidence levels: core (genes obtained from RefSeq/Genbank records – representing 7947 Ensembl genes), extended (supported by EST or partial mRNA evidence – an additional 6221 genes) and full (bioinformatically predicted – another 3650 genes). For this study we used the probes assigned with core and extended confidence levels, since this led to the detection of the largest number of DE genes between SNT and naive samples after correcting for multiple testing.

RNA-seq can, in theory, interrogate the transcription of any of the 26405 Ensembl genes through the mapping of the sequenced reads to their genomic location. It should be mentioned that these annotated Ensembl genes are not necessarily protein-coding genes; they include miRNAs, ribosomal RNAs and pseudo-genes, and will not necessarily be expressed in the DRG, nor selected for by poly(A) enrichment. In addition, a number of genes cannot be interrogated in practice because all of their exons overlap exons from other genes in terms of genomic location (either on the same strand, or opposite strands). Excluding genes for these reasons reduces the total number of genes detectable by our sequencing protocol to 26172 genes. Table 2 shows the overlap between the different platforms using the core, extended and full confidence Affymetrix probe annotations for the intersection of Ensembl genes annotated in both Rn4 and Rn5 genome builds.

**Table 2:** Total number of genes measurable by RNA-seq and exon arrays at the three probeset confidence levels investigated, for Ensembl ids found in both Rn4 and Rn5 genome builds

**Core confidence probesets**		**RNA-seq:**		**Totals**
		**Detectable**	**Not detectable**	
Microarray:	**Detectable**	7153	5	**7158**
	**Not detectable**	13638	118	**13756**
	**Totals**	**20791**	**123**	**20914**
**Core and extended confidence probesets**		**RNA-seq:**		**Totals**
		**Detectable**	**Not detectable**	
Microarray:	**Detectable**	13042	13	**13055**
	**Not detectable**	7749	110	**7859**
	**Totals**	**20791**	**123**	**20914**
**Core, extended and full confidence probesets**		**RNA-seq:**		**Totals**
		**Detectable**	**Not detectable**	
Microarray:	**Detectable**	15492	26	**15518**
	**Not detectable**	5299	97	**5396**
	**Totals**	**20791**	**123**	**20914**

Overlap between genes measurable by either platform, for all core probesets only (top table), core + extended probesets (middle table-the set of probesets used in this study), and core + extended + full probesets (bottom table).

To conclude, regardless of probe confidence levels, the RNA-seq protocol is potentially able to quantify a much larger number of genes than microarrays.

### Comparison of exon arrays and RNA-seq for the measurement of absolute gene expression

In order to compare the ability of the different platforms to detect gene expression we considered the genes that could be measured by both platforms (using core and extended confidence probesets). This amounts to a total of 13042, consisting of all the genes measurable by exon arrays excluding only those that contain overlapping exons and therefore cannot be measured by RNA-seq, and that are annotated in both the Rn4 and Rn5 genomes.

**Figure 4:**
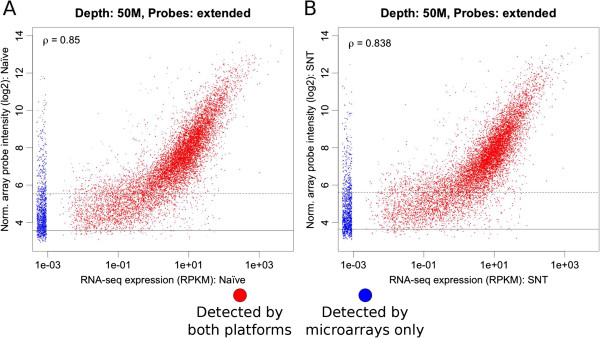
**Comparison of RNA-seq and microarrays for the measurement of gene expression.** Correlation between normalised hybridisation intensity and normalized read counts (RPKM) at a 50 M read depth for genes measureable using microarrays and RNA-Seq. **A)** Average expression for all three SNT samples. **B)** Average expression for all three naive samples. The red points show genes for which some expression level is measured by both platforms, blue points show genes that are not detected by RNA-seq (i.e. 0 reads aligned to the exons for that gene). Lines show the median (normalised) intensity for the antigenomic control probesets (solid line) and median + 1 median absolute deviation (dashed line). Some noise has been added to the expression values of these genes for clearer visualization of the point density.

Figure 4A depicts the correlation between the log_2_ normalised probe intensities for each transcript cluster and its respective expression level as determined by RNA-seq measured in reads per kilobase per million mapped reads (RPKM) as proposed by Mortazavi et al. [44]. There is a positive correlation between the hybridization intensities and RPKM for genes detected by RNA-seq (red points). However there are a number of genes not detected by RNA-seq (0 reads aligned to the exons for that gene; blue points). The two platforms show a large agreement for genes with high levels of expression, however there is less agreement for genes with low levels of expression. For example, for genes with a log normalised hybridization intensity value below ~6 in the exon arrays, RNA-seq is able to assign a much wider range of expression values.

Possible causes for the reduced correlation for more lowly expressed genes may be related to non-specific binding to probes for the microarrays or sampling error for RNA-seq, which would affect lowly expressed genes to a greater extent. In support of the non-specific binding for microarrays cause, we notice that there is very little correlation for the genes below the horizontal lines – these lines represent the median (solid line) and median plus one median absolute deviation (dashed line) values for the antigenomic control probes of the exon array. These probesets comprise probes that have been chosen to estimate background hybridisation; they do not match any sequence in the rat, mouse or human genome. Interestingly, there is a higher Spearman’s correlation coefficient between platforms for gene expression measurements of naive tissue (Figure 4A) than SNT (Figure 4B). The reasons for this are unclear, but could be related to the abilities of the different platforms to measure RNA expression in injured tissue. We have depicted the correlation plot for the 50 M read depth, as the results are qualitatively equivalent at lower read depths (Additional file 1).

### Comparison of exon arrays and RNA-seq for the detection of differential gene expression

**Figure 5:**
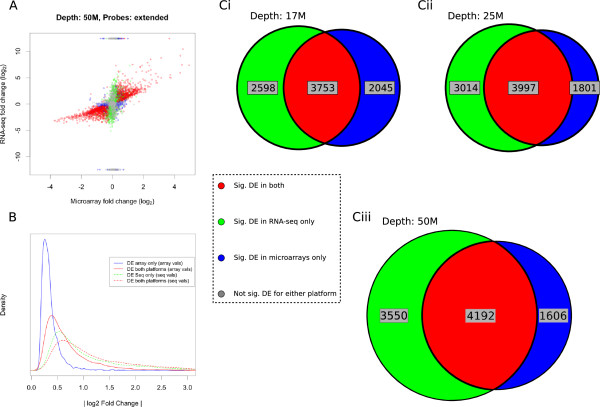
**Comparison of RNA-seq and microarrays for the detection of differentially expressed genes. A)** Correlation between fold changes estimated by microarrays and RNA-seq (50 M read depth) for genes detectable by both technologies. There is an overall concordance in direction of fold change for the genes deemed as significantly DE by both platforms (red points), however a large number of DE genes are detected exclusively by RNA-seq (green points) or microarrays (blue points). **B)** Plot of the distributions of absolute log_2_ FCs for DE genes. FCs are shown for the genes that are called as DE by both platforms (red lines, dashed and solid lines show RNA-seq and microarray fold changes), using RNA-seq only (dashed green line) and microarrays only (solid blue line). Distribution curve computed using the probability density function, implemented in R. **Ci-iii**) Venn diagrams showing the number of genes found to be differentially expressed by RNA-seq at distinct read depths (Ci- 17 M; Cii- 25 M; Ciii-50 M) and the overlap with microarray data.

We compared the ability of both platforms to detect differential gene expression. We compared log_2_ fold change (FC SNT/naive) values as determined by RNA-seq to exon arrays. We observed a general agreement in the direction of FC for significantly DE genes detected by both platforms (Figure 5A, red points), with some DE genes being exclusively detected by RNA-seq (green points). Interestingly, a number of genes not detected by RNA-seq in one of the experimental groups – giving rise to infinite FCs (blue points) – are deemed significantly DE by exon arrays. However the direction of FCs does not necessarily agree between the two platforms and these genes usually show a small fold-change value. This suggests that for some genes, the apparently significant FC detected by exon arrays may be due to the effects of non-specific binding, or some general technical variability, although in some cases this might be due to the RNA-seq protocol not being able to align enough reads to classify the gene as significantly DE. Further experimental validation of these genes is necessary in order to confirm that they are truly changing between samples, but this change cannot be detected using RNA-seq.

It is also notable that a far wider range of log_2_ FC is detectable using RNA-seq. This is shown in Figure 5B, which shows the distribution of absolute log_2_ FCs found by either technology. In addition, we can see that the genes called DE only using RNA-seq show a similar distribution of FCs to the genes called DE by both platforms. Conversely the genes called DE only using microarrays show a much narrower distribution of FCs than the genes called DE by both platforms. Additional file 2 shows the distributions of FCs for significantly DE genes alongside those genes that are not called DE by either platform. The significantly DE genes exclusively detected by microarrays show a similar distribution to the non-DE microarray genes. Conversely, the separation between the distributions of FCs for the DE RNA-seq and the non-DE RNA-seq genes is much clearer. This supports the idea that some of the genes detected as DE by microarray only are likely to be called as such because of noise related to non-specific binding. This leads to a small but consistent FC between samples and not true transcriptional changes.

Figure 5C shows the overlap between the DE genes found by microarrays and RNA-seq at distinct read depths. We see a large overlap between the different platforms; this overlap increases with RNA-seq depth. We also see that a higher sequencing depth leads to a sharp increase in the number of genes that are called DE using RNA-seq only.

### Comparison of exon arrays and RNA-seq at the exon level

There are 233498 exons annotated in the rat genome according to Ensembl (version 69). Removing overlapping exons leaves 209219 that can be probed uniquely by RNA-seq. Microarrays can probe 146765 exons (using core and extended confidence probesets). In order to compare the two platforms directly, in terms of expression of individual exons, RNA-seq reads were aligned to the Rn4 genome assembly for this analysis (Additional file 3). As with Figure 5A, we see a good agreement between platforms. However the correlation is less strong for exons than for genes. Reasons for the weaker correlation include the increased sampling error inherent when counting reads mapping to exons, since exons represent a much shorter genomic area than genes. Estimating microarray expression at the exon level is also likely to be less accurate than estimating gene expression, due to the reduced number of probes across which to summarise the probe intensity value. A weaker correlation between platforms is also observed for FCs between SNT and naive samples (Additional file 3: Figure B). The Venn diagram in Additional file 3: Figure C shows that at a 50 M read depth microarrays and RNA-seq detect a similar amount of differentially expressed exons. This is in contrast with the gene level comparison, which shows that RNA-seq can detect a much higher number of DE genes. Both of these observations are likely to be due to sampling error, leading to difficulties in separating RNA-seq expression from “shot noise” using the DESeq algorithm (as described in [19]). Sequencing to a higher depth could reduce this sampling error. Because the exons are much shorter, shot noise is more problematic at the exon level than the gene level. We also notice that for lower sequencing depths the number of exons called DE using RNA-seq is much lower, suggesting 50 M is the minimum read depth that should be used when investigating DE of exons in heterogeneous tissue (Additional file 4).

### Sequencing depth and the detection of differentially expressed genes

**Figure 6:**
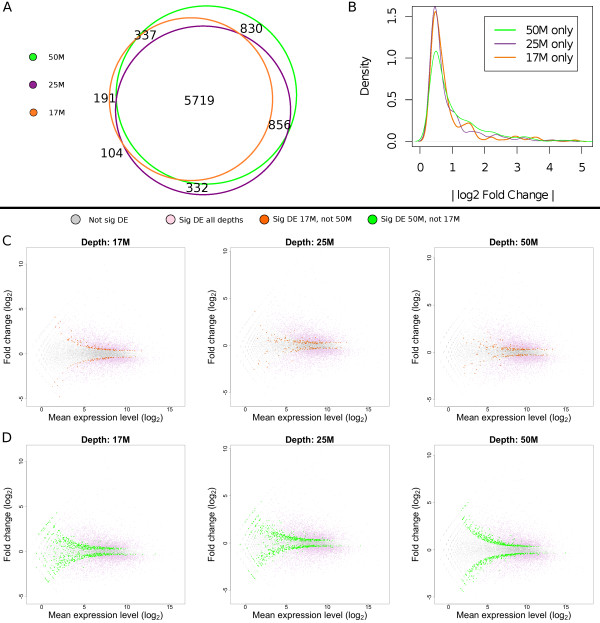
**Sequencing depth and the detection of differentially expressed genes. A)** Venn diagram showing the overlap between the total numbers of DE genes found at the different sequencing depths. **B)** Distributions of absolute log_2_ fold changes for the DE genes found at each sequencing depth. **C)** The 194 genes deemed as DE exclusively at 17 M read depth (but not at higher read depth), plotted as log_2_ read count vs. log_2_ fold change at three distinct read depths (orange points), along with not significantly DE genes (grey points) and genes significantly DE at all read depths (pink points). As read depth increases, the estimated fold changes for genes with low mean read count decreases, suggesting that the estimation of DE at lower sequencing depths suffers from sampling errors for genes with low read count. **D)** More plots of log_2_ read count vs. log_2_ fold change for all genes at all three respective sequencing depths. Genes detected as DE exclusively at a 50 M read depth (896 genes in total) are shown as green points.

We investigated the effect of sequencing depth on the ability of RNA-seq to find differentially expressed genes. In order to do this we sequenced replicates of each sample at three depths: 17 M, 25 M and 50 M reads. The Venn diagram in Figure 6A shows the total number of DE genes detected at each depth. It is clear that, whilst there is a very large overlap between the results at all three sequencing depths, increasing read depth leads to the detection of a higher number of DE genes. Generally, genes detected as DE at a lower sequencing depth will also be detected as DE when sequencing to higher depth. However it should be noted that a small number of genes are detected as DE at lower sequencing depths only.

We considered the differential expression of genes that were detected as DE at a depth of either 17 M reads only, or 50 M reads only. Figure 6B shows the distribution of absolute log_2_ FCs for these genes. The genes found DE exclusively at a 50 M read depth show a wider range of FCs, with fewer genes showing a log 2 fold change less than one. This observation is likely to be related to sampling errors for genes with a low read count, which have a higher impact on measurements at low read depths: through sampling error, these genes may have obtained inflated (higher) values for SNT samples and deflated (lower) values for naive samples, or vice-versa, when in reality there is no change between samples. This is often a problem for RNA-seq experiments, since limitations related to the cost of the technology often mean that expression can only be measured for a limited number of samples.

To further investigate the hypothesis that the DE genes found at a depth of 17 M may be erroneously labelled as DE due to sampling error, we have plotted the mean number of aligned reads against FC for all genes (Figure 6C). The 295 genes deemed as DE at 17 M only are highlighted in orange. We see that the 17 M only genes are on the cusp of significance at a depth of 17 M, and that many have low read counts, i.e. few reads align to these genes. In addition, many of these genes show much lower FCs at a depth of 25 M and 50 M reads.

The opposite is true for the genes deemed significant at a depth of 50 M only (highlighted in green in Figure 6D) – we see that, at all sequencing depths, most of these genes still maintain large FCs, and are situated close to the pink coloured points, which represent the genes significant at both 17 M and 50 M read depths.

In summary, using the higher sequencing depth of 50 M reads leads to the detection of a larger number of DE genes, particularly for genes with low read counts.

### Intronic expression and its effect on fold change calculation

We also examined expression from intronic regions, a feature of expression that can be quantified much more precisely and comprehensively when using RNA-seq than exon arrays, which by definition are designed to probe known (and predicted) exons.

**Figure 7:**
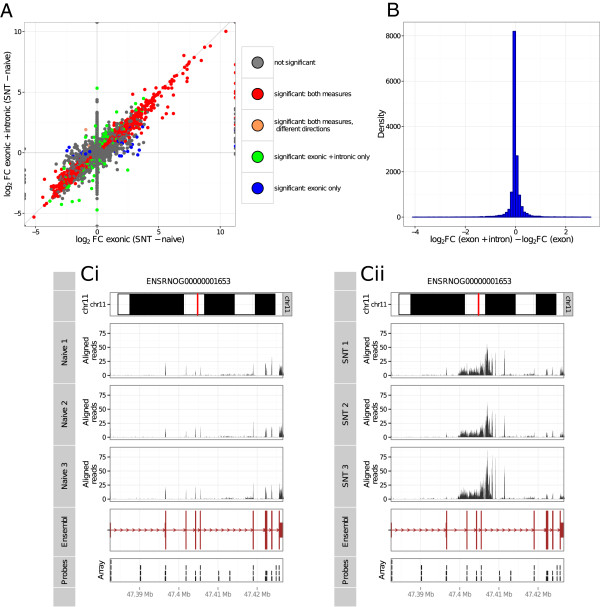
**The effect of intronic expression on fold change calculation. A)** Estimation of log_2_ FC considering exonic reads only (x-axis) compared to FC calculating counting exonic and intronic reads (y-axis). Red points represent genes called DE when using both counting schemes with the same direction of fold change, peach points represent the two genes that are called as DE with both schemes, but with opposite directions of fold change. Green points show genes called DE when considering exonic reads only, but not when considering exonic and intronic reads. Blue points show genes DE when considering exonic and intronic reads but not when considering exonic reads only. **B)** Distribution of the ratio of fold changes estimated by both methods. Calculated by subtracting the log_2_ FC values calculated using full gene expression from log_2_ FC calculated exon expression only. **Ci, Cii)** Genome graphs for gene St3gal6, showing intronic expression that is not proportional to exonic expression, i.e. that is increased following SNT. The figure comprises a series of “tracks” for each gene, and its expression levels for SNT samples (Ci) and naive samples (Cii). The top tracks show the genomic coordinates of the gene on chromosome 11 (precise position marked in red). The middle histogram-like tracks show the positions of RNA-seq reads mapping to the genomic location of the gene. Below these tracks is a track showing the gene structure (exons are represented by boxes, introns are represented by arrowed lines, the direction of these arrows shows the direction of transcription). Bottom track shows the position of the microarray probes that map to the genomic location of the gene.

In Figure 7A we compare the log_2_ FCs in gene expression calculated when considering exonic reads only (x axis) with log_2_ FCs calculated considering exonic + intronic reads (y-axis). If intronic expression is absent or is proportional to exonic expression in all samples then the point that represents a given gene will fall close to x = y on the graph. This is the case for the majority of genes (Figure 7B). However there are also a number of genes where intronic expression is proportionally much higher for SNT samples than naive samples, and vice versa. This change in fold change as a result of including reads that align to intronic regions may reflect the inclusion of novel exons in these transcripts, may be due to the expression of unannotated genes that occur at the same loci, or possibly due to non-coding RNAs. We measured the significance of these relative changes in intronic expression between naive and SNT samples using DEXSeq [45] (Additional files 5 and 6). Using an FDR cutoff of 0.1, we found 2030 genes showing a significant change in ratios of intronic to exonic expression following SNT (1914 genes show a relative increase in expression from non-exonic regions following SNT, whilst 116 of these genes show a decrease), these results were similar at more conservative FDR cutoffs. In Figure 7C we show an example of one of the top genes found using this method (in terms of a low p-value): ST3 beta-galactoside alpha-2,3-sialyltransferase 6 (St3gal6). We show genome graphs for the genomic coordinates of the gene, showing expression in the naive samples (left, 7 Ci) and SNT samples (right, 7Cii). We see that for SNT samples more reads map to intronic regions, and less to exonic regions. This results in contrasting patterns of differential expression for this gene after SNT: downregulation when only exonic reads are considered, and upregulation if exonic + intronic reads are considered. These reads may represent novel exons of the annotated St3gal6 gene that are expressed after SNT; alternatively, they may originate from a yet unannotated gene with overlapping genomic coordinates.

In any case, the pattern of expression of the transcript (or transcripts) arising from this genomic location after SNT, demonstrates an advantage of RNA-seq: finding areas of expression occurring outside of annotated exons. Using microarrays, we would not be able to find such unusual intronic expression, as shown by the positions of exon array probes for the genes in Figures 7C: although exon arrays do sometimes probe intronic regions, we see the coverage is not as comprehensive as that offered by RNA-seq. The biological significance of non-exonic expression is still very much an open question, however it is clear that over the last few years more and more such regions of non-exonic expression have being detected and several functions have being ascribed to them [42,46].

### Biological function analysis

We compared two genome-wide expression technologies in their ability to detect differential gene expression in L5 DRGs in response to SNT. Having established that RNA-seq outperforms exon arrays from a technical point of view, we investigated how the biological insights provided by the two datasets compare. Firstly, we interrogated the datasets for differential expression of classic “pain markers” as well as novel candidate genes, and secondly we performed functional analysis of entire datasets to investigate physiological, cellular and molecular events that are disturbed by SNT and may underlie pain conditions.

A number of published microarray studies have addressed gene expression changes in models of pain [1,4,34]. Genes typically dysregulated in pain conditions include injury markers (e.g. Atf3), ion channel subunits (e.g. Cacna2d1, Kcnc2), neuropeptides (Gal, Npy), inflammatory mediators such as cytokines and chemokines (Ccl2, Cxcl10, Cxcl13), and growth factors (Vgf). LaCroix-Fralish et al. [4] performed a systematic review of microarray studies in rodent neuropathic and inflammatory pain models, identifying a list of genes that are commonly dysregulated. In our exon array dataset, the great majority of these genes appeared dysregulated in the direction expected (Table 3). Reassuringly, this was in agreement with RNA-seq data, with the added advantage that the magnitude of FC estimated for the same genes was higher in RNA-seq, a reflection of its higher dynamic range.

**Table 3:** Differential gene expression of commonly dysregulated genes in experimental pain models

**Gene Symbol**	**Gene name**	**Fold change RNA-Seq**	**Fold change exon arrays**
**Genes upregulated after SNT**			
Aif1/Iba-1	Allograft inflammatory factor 1 (Iba-1)	4.7	2.0
Apoe	Apoliprotein E	1.5 (ns)	1.2
Arg1	Arginase, liver	30.1	2.4
Arpc1b	Actin related protein 2/3 complex, subunit 1B, 41 kDa	3.7	2.7
Atf3	Activating transcription factor 3	33.8	13.7
C1qb	Complement component 1, q subcomponent, B chain	10.1	5.5
C1qc	Complement component 1, q subcomponent, C chain	7.7	4.5
C1s	Complement component 1, s subcomponent	4.4	2.5
Cacna2d1	Calcium channel, voltage-dependent, alpha 2/delta subunit 1	5.0	3.0
Ccl2	Chemokine (C-C motif) ligand 2	2.1	1.4
Ccnd1	Cyclin D1	4.1	2.7
Cd74	CD74 molecule, major histocompatibility complex, class II invariant chain	6.5	2.8
Coro1a	Coronin 1-A	1.0 (ns)	1.2 (ns)
Crabp2	Cellular retinoic acid-binding protein 2	3.1	2.1
Csrp3	Cysteine and glycine-rich protein 3 (cardiac LIM protein)	590.2	22.6
Ctsd	Cathepsin D precursor	1.4 (ns)	1.3
Ctsh	Cathepsin H	1.6	1.3 (ns)
Cxcl10	Chemokine (C-X-C motif) ligand 10	7.5	3.8
Cxcl13	Chemokine (C-X-C motif) ligand 13	4.0	2.2
Egr1	Early growth response 1	2.2	1.8
Gabra5	Gamma-aminobutyric acid (GABA) A receptor, alpha 5	2. 5	2.1
Gadd45a	Growth arrest and DNA-damage-inducible, alpha	6.8	4.6
Gal	Galanin/GMAP prepropeptide	46.3	13.5
Gap43	Growth associated protein 43	3.2	2.3
Gfap	Glial fibrillary acidic protein	8.8	3.8
Gfra1	GDNF family receptor alpha 1	3.2	2.1
Igfbp3	Insulin-like growth factor binding protein 3	4.7	2.9
Igfbp6	Insulin-like growth factor binding protein 6	1.8	1.5
Lum	Lumican	2.5	1.6
Npy	Neuropeptide Y	Not detected	7.8
Reg3b	Regenerating islet-derived 3 beta	61.0	20.1
S100a4	S100 calcium binding protein A4	2.8	1.9
Sprr1a	Small proline-rich protein 1A/cornifin-1	176.6	57.9
Stmn4	Stathmin-like 4	6.1	3.2
Timp1	TIMP metallopeptidase inhibitor 1	3.5	2.1
Vgf	VGF nerve growth factor inducible	5.3	2.5
Vip	Vasoactive intestinal peptide	138.1	5.4
**Genes downregulated after SNT**			
Atp1b3*	ATPase, Na+/K + transporting, beta 3 polypeptide	0.6	0.8
Calca*	Calcitonin-related polypeptide alpha	0.3	0.4
Cd55	CD55 molecule, decay accelerating factor for complement	0.2	0.3
Chrna3	Cholinergic receptor, nicotinic, alpha 3 (neuronal)	0.1	0.1
Ckmt1	Creatine kinase, mitochondrial 1, ubiquitous	0.2	0.3
Gabbr1	Gamma-aminobutyric acid (GABA) B receptor, 1	0.8	0.8 (ns)
Grik1	Glutamate receptor, ionotropic, kainate 1	0.2	0.1
Htr3a	5-hydroxytryptamine (serotonin) receptor 3A, ionotropic	0.1	0.1
Kcnc2	Potassium voltage-gated channel, Shaw-related subfamily, member 2	0.3	0.5
Nefh	Neurofilament, heavy polypeptide	0.3	0.4
Nefl	Neurofilament, light polypeptide	0.2	0.5
Nefm	Neurofilament, medium polypeptide	0.3	0.5
Nsf	N-ethylmaleimide-sensitive factor	0.5	0.5
Rab3a	RAB3A, member RAS oncogene family	0.3	0.4
Rgs4	Regulator of G-protein signaling 4	0.2	0.2
Scn11a	Sodium channel, voltage-gated, type XI, alpha subunit	0.1	0.1
Snap25	Synaptosomal-associated protein, 25 kDa	0.3	0.6
Sst*	Somatostatin	0.1	0.1
Sv2b	Synaptic vesicle glycoprotein 2B	0.3	0.3
Tac1*	Tachykinin, precursor 1	0.3	0.3
Vsnl1	Visinin-like 1	0.2	0.3
Ywhag	Tyrosine 3-monooxygenase/tryptophan 5-monooxygenase activation protein gamma polypeptide	0.5	0.7

The list of genes resulted from a meta-analysis study of microarray data of DRG and/or spinal cord tissue in inflammatory and neuropathic pain models [4]. Fold changes expressed as ratio SNT/naive in L5 DRGs. All fold changes are significant (p < 0.1, FDR) except if indicated by “ns” – non significant. The direction of fold change is consistent between the exon array and RNA-Seq dataset and largely coincides with the reported trends. Exceptions are genes marked with “*” Atp1b3, Calca, Sst, Tac1 which are listed as upregulated in the meta-analysis study but are significantly downregulated in our study. In support of our results, qPCR data reported by LaCroix-Fralish et al. [4] suggested that these genes are down regulated (albeit not significantly) in DRG tissue after chronic constriction injury. Also Npy expression is not detected in RNA-seq because there is a paralogous gene to Npy sharing 98% sequence homology. Therefore, reads aligning to Npy would be deemed as ambiguous and discarded from our analysis. Mapping to the Rn4 assembly of the rat genome (where paralogous genes are not annotated) reveals a 36.4 upregulation of Npy.

In high throughput transcriptomic studies, the prioritization of candidates for further validation is generally dictated by the magnitude (and significance) of FC. In order to determine how the choice of candidates is influenced by technological platform, we compiled lists of the top 50 significant fold changes for each method (Table 4). Twenty-five genes are simultaneously ranked amongst the top 50 by both techniques. For the remaining genes in the RNA-seq top 50, thirteen are also deemed as DE by exon arrays, although with a lower FC, two genes are not deemed significantly dysregulated by exon arrays and ten genes cannot be measured/detected by exon arrays due to lack of probes at the core or extended confidence levels. Amongst the top 50 genes found by exon arrays that do not coincide with the RNA-seq top 50, twenty-two are still deemed as DE by RNA-seq (with fold changes higher than 4 fold). The remaining three genes cannot be detected by our RNA-seq alignment protocol, due to the existence of paralogous genes with high sequence conservation, which leads to reads from these transcripts being classified as ambiguous and discarded from the analysis.

**Table 4:** Top 50 significantly upregulated genes in RNA-seq and exon arrays

**Gene symbol**	**Gene name**	**Rank RNA-seq**	**Rank exon arrays**	**Fold change RNA-seq**	**p adj RNA-seq**	**Fold change exon arrays**	**p adj exon arrays**
Crisp3	Cysteine-rich secretory protein 1	1	5	1414.6	7.06E-10	21.0	2.58E-03
Csrp3	Cysteine and glycine-rich protein 3	2	3	590.1	4.30E-44	22.6	8.59E-05
Mmp12	Macrophage metaloelastase	3	14	427.5	2.98E-03	7.8	1.58E-02
Tgm1	Protein-glutamine gamma-glutamyltransferase k	4	25	355.1	2.18E-43	5.9	1.39 E-03
Hpd	4-hydroxyphenylpyruvate dioxygenase	5	34	237.3	1.56E-20	4.9	1.11E-03
Ucn	Urocortin	6	187	180.1	2.92E-36	2.5	1.10E-03
Sprr1a	Small proline-rich protein 1a / Cornifin-a	7	1	176.6	2.02E-25	57.9	2.36E-05
Serpina3n	Serine protease inhibitor A3N	8	4	174.6	1.97E-44	21.6	3.66E-04
Cxcl14	Chemokine (C-X-C motif) ligand 14	9	2	167.3	3.62E-08	33.4	1.59E-03
Hamp	Hepcidin antimicrobial peptide	10	11	162.5	5.41E-35	13.1	1.11E-03
Ptprh	Receptor-type tyrosine-protein phosphatase h	11	49	161.4	2.48E-48	3.9	1.40E-04
Rgd1305807 / LOC298077	Uncharacterized protein	12	2333	159.3	6.66E-06	1.2	1.89E-02
Cldn4	Claudin 4	13	31	159.2	3.29E-42	5.3	1.391E-03
Mmp7	Matrix metallopeptidase 7	14	319	158.3	2.62E-02	2.0	3.85E-02
Vip	Vasoactive intestinal peptide	15	28	138.2	3.21E-25	5.4	1.39E-03
Mroh4	Maestro heat-like repeat family member 4	16	ND	132.8	3.58E-08	-	-
Stac2	SH3 and cysteine-rich domain-containing protein 2	17	46	126.0	2.35E-69	4.1	1.43E-03
Ucn2	Urocortin 2	18	21	88.8	5.08E-04	6.0	2.67E-03
Il6	Interleukin 6	19	41	78.4	8.66E-18	4.5	1.58E-03
Serpinb2	Plasminogen activator inhibitor 2 type a	20	95	75.7	1.09E-34	3.2	6.07E-03
Abp1	Amiloride-sensitive amine oxidase	21	2050	73.8	6.06E-08	1.3	1.89E-02
D3zu79_rat/ lsmem1	Leucine rich single pass membrane protein	22	ND	73.7	8.13E-07	-	-
Ankrd1	Ankyrin repeat domain-containing protein 1	23	40	68.3	5.46E-31	4.5	2.22E-03
Cd8b	T-cell surface glycoprotein cd8 beta chain precursor	24	105	67.4	1.75E-02	3.0	1.24E-02
Vtcn1	V-set domain containing t cell activation inhibitor 1	25	183	67.4	1.13E-30	2.5	4.33 E-04
RT1-M2	RT1 class IB, locus M2	26	ND	64.9	2.54E-08	-	-
Il1a	Interleukin-1 alpha precursor	27	642	64.2	3.14E-22	1.7	5.58E-03
Reg3b	Regenerating islet-derived protein 3-beta	28	6	61.0	1.45E-114	20.1	2.36E-05
Il24	Interleukin-24	29	19	55.3	3.21E-25	6.4	1.39E-03
Igsf23	Immunoglobulin superfamily, member 23	30	ND	53.7	2.41E-28	-	-
En1	Homeobox protein engrailed	31	ND	51.6	2.90E-18	-	-
Trim55	Tripartite motif-containing protein 55	32	2142	49.4	1.24E-02	1.3	4.16E-02
Igsf7	Immunoglobulin superfamily, member 7	33	ND	46.9	1.51E-02	-	-
Cd8a	T-cell surface glycoprotein CD8 alpha chain	34	20	46.7	2.16E-03	6.1	4.13E-03
Gal	Galanin/GMAP prepropeptide	35	10	46.3	3.94E-58	13.5	1.36E-04
LOC363060/ Plet1	Placenta-induced transcript 1	36	588	41.2	5.17E-08	1.7	4.41E-03
Vsig4	V-set and immunoglobulin domain containing 4	37	1752	39.7	1.18E-02	1.3	5.80E-02
Nps	Neuropeptide S	38	ND	37.4	2.07E-07	-	-
Htr2b	5-hydroxytryptamine receptor 2b, g-protein coupled	39	18	36.3	8.44E-04	7.2	3.89E-03
Col7a1	Collagen alpha-1(VII) chain precursor	40	1426	34.2	2.37E-11	1.4	1.60E-02
Atf3	Activating transcription factor 3	41	9	33.8	2.36E-66	13.7	1.99E-05
Novel	Novel protein coding	42	ND	32.8	1.61E-03	-	-
Gpnmb	Transmembrane glycoprotein nmb	43	8	31.9	4.00E-03	14.3	1.39E-03
Lilrb4	Leukocyte immunoglobulin-like receptor, subfamily b, member 4	44	12	31.6	6.33E-02	9.1	3.14E-02
Gzmb	Granzyme b (granzyme 2, cytotoxic t-lymphocyte-associated serine esterase 1)	45	7	31.4	2.52E-11	16.6	4.87E-05
Arg1	Arginase-1	46	197	30.1	2.30E-66	2.4	2.00E-03
Fcrls	Fc receptor-like s, scavenger receptor	47	ND	30.0	7.34E-05	-	-
Mmp10	Matrix metallopeptidase 10	48	NS	29.2	4.47E-03	1.1	NS
Lce1f	Late cornified envelope 1F	49	ND	28.3	5.03E-04	-	-
Cnga4	Cyclic nucleotide gated channel alpha 4	50	NS	27.7	5.20E-12	1.1	NS
Npy	Neuropeptide Y	ND	13	-	-	7.8	4.12E-03
Cthrc1	Collagen triple helix repeat containing 1	90	15	16.9	4.57E-04	7.6	2.39E-03
Clec7a	C-type lectin domain family 7, member a	91	16	16.2	5.58E-04	7.4	3.61E-03
Cd68	Macrosialin precursor	71	17	21.8	4.51E-03	7.4	3.84E-03
Thbs2	Thrombospondin 2 precursor	87	22	17.3	1.66E-04	6.0	3.41E-03
Ccl9	Chemokine (C-C motif) ligand 9	55	23	25.3	8.48E-07	5.9	4.92E-03
Apobec1	Apolipoprotein B mRNA editing enzyme, catalytic polypeptide 1	104	24	14.3	1.37E-04	5.9	4.62E-03
C1qb	Complement C1Q subcomponent subunit B precursor	160	26	10.1	2.82E-04	5.5	2.15E-03
Cdkn1a	Cyclin-dependent kinase inhibitor 1	174	27	9.6	1.20E-29	5.4	5.12E-04
Postn	Periostin precursor	132	29	11.3	1.33E-04	5.4	4.78E-03
Fcgr2a	Low affinity immunoglobulin gamma fc region receptor iii	ND	30	-	-	5.3	4.26E-03
Crlf1	Cytokine receptor-like factor 1	110	32	13.1	4.06E-04	5.1	4.22E-03
C1qa	Complement C1Q subcomponent subunitA	135	33	11.2	9.39E-05	5.0	1.43E-03
Trem2	Triggering receptor expressed on myeloid cells 2	68	35	22.3	2.64E-03	4.8	7.16E-03
Cxcl9	Chemokine (C-X-C motif) ligand 9	301	36	6.7	7.44E-09	4.6	5.39E-03
Socs3	Suppressor of cytokine signaling 3	239	37	7.7	1.66E-06	4.6	1.09E-02
Gadd45a	Growth arrest and DNA damage-inducible protein gadd45 alpha	287	38	6.8	5.03E-25	4.6	3.03E-04
C1qc	Complement c1q subcomponent subunit c	235	39	7.7	2.65E-04	4.5	2.99E-03
Ly49si2	Immunoreceptor ly49si2	ND	42	-	-	4.5	2.92E-02
RT1-DA	RT1 class II, locus Da	307	43	6.7	5.88E-17	4.3	2.45E-03
Tgfbr1	Transforming growth factor, beta receptor 1	528	44	4.6	5.23E-03	4.2	4.92E-03
Ecel1	Endothelin converting enzyme-like 1	51	45	27.0	1.52E-93	4.1	4.87E-05
Cx3cr1	Chemokine (C-X3-C motif) receptor 1	219	47	8.1	1.08E-05	3.9	3.40E-03
RT1-BB	RT1 class II, locus Bb beta chain	308	48	6.7	5.23E-06	3.9	1.76E-03
Cxcl10	Chemokine (C-X-C motif) ligand 10	251	50	7.5	5.36E-03	3.8	1.88E-02

Rank indicates highest significant fold changes determined by each method in descending order. In order to obtain a numeric FC for genes with infinite fold changes, a read count of one was ascribed to the naïve samples.

NS- Non significant; ND- not detectable by exon arrays due to lack of probes in the core or extended confidence level or not detectable by RNA-Seq due to the existence of paralogous rat genes sharing high sequence homology leading to reads being classified as ambiguous and discarded from the analysis.

**Figure 8:**
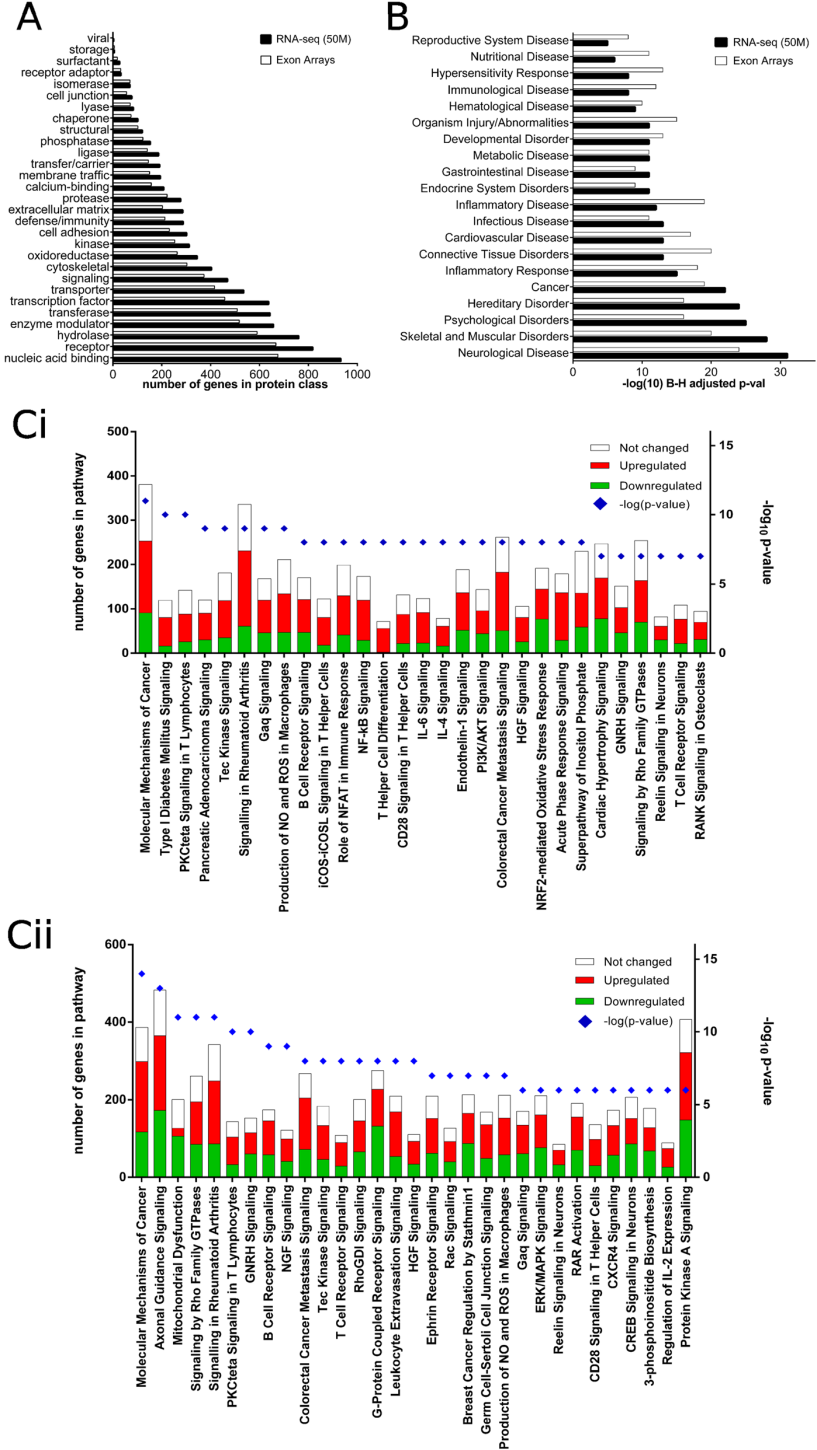
**Functional analysis of differentially expressed genes after SNT as determined by RNA-seq (50 M) and exon arrays. A)** Distribution of DE genes according to respective protein classes is similar for both datasets. **B)** Top Biological Functions/“Diseases and Disorders” assigned to DE genes largely overlap between the two datasets. **Ci, Cii)** Statistically overrepresented “canonical pathways” rank differently between the datasets, with top pathways in exon arrays being mostly related to immune function (Ci), while in RNA-seq, neuronal pathways are more represented (Cii).

The knowledge generated by our high throughput studies is not restricted to the identification of individual candidate genes in pain. Gene ontology and literature information on the roles of dysregulated genes provide insights into the biological phenomena compromised after SNT. As depicted in Figure 8A, the distribution of dysregulated genes based on corresponding “protein classes” (as categorized by PANTHER) is similar in both datasets.

Similarly, functional analysis using Ingenuity Pathway Analysis (IPA) revealed similar significant enrichment for the same biological functions/“diseases and disorders” for both datasets (Figure 8B). As expected, and in agreement with previously published studies [47-49], categories related to “neuronal/neurological function” and “immune/inflammatory” are significantly enriched.

Despite the overall similarity in top biological functions, a more detailed analysis using “canonical pathways”, annotated in IPA revealed very specific features of each of the datasets. While the most significant physiological/cellular/molecular pathways in the exon array dataset reflect the heavy contribution of immune system-related genes to the overall profile of the tissue after injury (Figure 8 Ci), in the RNA-Seq dataset there is a clear representation of neuronal pathways (Figure 8 Cii). These include “axonal guidance signalling”, “ephrin receptor signalling”, “Nerve Growth Factor (Ngf) signalling”, “reelin signalling in neurons”, and “CREB-signalling in neurons”. The overrepresentation of neuronal pathways in the RNA-seq dataset is due to the number of genes assigned to these pathways that are deemed as DE by RNA-seq but not by exon arrays. For example, a total of 483 genes are ascribed to the “axon guidance” pathway according to the IPA “canonical pathways” database. In our datasets, 292 “axon guidance genes” are simultaneously deemed as DE by microarrays and RNA-seq. However an additional 73 genes are exclusively detected by RNA-seq. Furthermore, the list of DE genes detected by RNA-seq only features molecules with established functions in nociception and/or pain such as neuronal nitric oxide synthase 1 (Nos1), and the transient receptor potential cation channel, subfamily V, member 4 (Trpv4, [50]), as well as a number of molecules belonging to protein categories such as “receptors”, “transporters”, “G-protein coupled receptors”, “ion channels” and “signalling molecules”, which may be important in neuronal function.

## Discussion

### RNA-seq has several advantages over microarrays

RNA-seq technology presents a novel tool for comprehensive, high throughput whole genome transcriptional profiling. In this study we profiled injured L5 DRG tissue following spinal nerve transection, using RNA-seq and microarrays. The same RNA samples were used for both platforms to enable a direct comparison of these technologies in an experimental pain model for the first time. We assessed both technologies for their ability to interrogate the transcriptome, detect gene expression, and to identify dysregulated genes that represent putative novel pain mediators.

Hammer and colleagues [9], have reported the use of RNA-seq to profile expression in the uninjured L4 DRG following L5 spinal nerve transection and reported a higher number of transcriptional changes in the SNT model than previously estimated in published microarray studies.

In the more direct comparison presented here, we also found a larger number of DE genes using RNA-Seq than using exon arrays, consistent with the literature [20,24,25]. This is partly due to the wider dynamic range of RNA-seq, as microarrays suffer from non-specific binding to probes, and signal saturation [13,20]. Non-specific binding leads to background signals which affect detection/quantification of lowly expressed genes, while highly expressed mRNA species may saturate the fluorescent signal, which can compromise the detection of differential expression. However it should be noted that estimation of DE for short length/lowly expressed genes can be also inaccurate for RNA-seq [18]. This is especially important with regards to exons – our study found a similar number of DE exons using either platform when sequencing to a depth of 50 M, and microarrays outperformed RNA-seq at lower sequencing depths (Additional file 3).

Discrepancy in the number of DE genes detected by either method may also be caused by the different analysis methods employed (limma for microarray analysis [51] and DESeq for RNA-seq [19]). However we believe that these do not have a significant impact on our findings, since both methods apply variance-shrinking steps to reduce false positives. In fact, DESeq has been shown to be one of the most conservative methods for RNA-seq differential expression detection [52]. The variance-shrinking steps are implemented to deal with small fold changes occurring by chance due to extremely low values of variance between samples, a common problem when testing a large number of genes across a small number of samples. Such shrinkage methods have been shown to outperform methods based on simple t-tests and fold change cut-offs [53]. For this reason we did not impose an “effect size filter” when determining significantly DE genes. Finally, we do not believe that the FDR cutoff (0.1) favoured either technology, as we obtained qualitatively similar results with other FDRs (Additional file 7).

Despite the previously mentioned advantages of RNA-seq over microarrays, we observed a number of genes detected as DE using microarrays but not classed as such by RNA-seq (Figure 5). We have shown in Figure 4B and Additional file 2 that most of these genes have low FC values. Some of these genes may be false positives: the apparent significant change in expression might be due to non-specific binding. However it is also likely that some of these genes are truly DE. There are several reasons why microarrays might detect DE genes that are missed when using RNA-seq. For example, some genes will share high homology with other genes or pseudo genes, thus making it difficult to map reads to the genes unambiguously, as observed for three genes listed in Table 4 that appear DE in exon arrays and are classified as non-detected by RNA-seq. This should not be a problem for highly expressed genes, as long as there are enough regions of unique sequence along the length of the gene to make a robust signal, but can be a problem for lowly expressed genes, or genes with repetitive regions. Another reason is that lowly expressed genes will be strongly affected by random sampling – the expression of a gene for which only 10 copies are present in a sample is much less likely to be estimated accurately than a gene for which 1000 copies are present. The obvious solution to this issue with RNA-seq is to increase the sequencing depth, enabling a more accurate estimation of lowly expressed genes in conjunction with the increased detection of differential expression.

### Functional analysis of DE genes reveals consistent results across datasets

Although RNA-seq detected a larger number of DE genes, functional enrichment analysis of the microarray and RNA-seq datasets individually using Ingenuity Pathway Analysis revealed qualitatively similar results. In agreement with previous studies in animal models of pain, functional categories/“diseases and disorders” related to immune function/inflammatory response, as well as neurological disease were statistically overrepresented in the respective lists of DE genes [47-49]. This suggests that the genes found exclusively with RNA-seq are likely to be true positives – as they fall into the categories already enriched amongst the overlapping genes. Had these genes arisen as an artefact of RNA-seq, we would expect less coherence in terms of enriched categories in the RNA-seq results, and reduced significance. This is consistent with our canonical pathway analysis where a number of neuron-specific pathways rank highly in the RNA-seq dataset (Figure 8 Cii), because ~10-20% of genes assigned to these pathways are deemed as DE by RNA-seq but not by exon arrays. In terms of discovery of putative pain mediators, RNA-seq has clear advantages over exon arrays as it unravels candidate genes that exon arrays (at the core and extended probeset confidence levels) would fail to identify (e.g. Nos1, Trpv4 and other molecules with established or putative functions in neurotransmission). In contrast, the great majority of putative candidates identified by exon arrays can be confidently identified by RNA-Seq.

In summary, our gene expression datasets accurately reflect the biological mechanisms triggered by peripheral nerve injury as demonstrated in prior pain and injury-related studies. However, it is worthwhile pointing out that our functional analysis is shaped by current literature information used to build the Ingenuity database and may also be incomplete due to lack of gene ontology annotations for a large proportion of genes in both datasets. We expect that the functional information provided by our dataset will become more refined as gene ontology and pathway annotations evolve.

### A higher sequencing depth leads to the detection of a greater number of DE genes

We compared three distinct sequencing depths (17 M, 25 M, 50 M reads/sample) in terms of the number of DE genes detected (Figure 5). Increasing RNA-seq read depth leads to the detection of a higher number of DE genes (Figure 5C). A recent study has shown that a high sequencing depth could lead to the false detection of genes that are not expressed in the sample [54]. However, it is unlikely that we have reached such a point; moreover, it is unclear whether reaching such a point would affect DE calculation in an experimental design that takes into account biological variation between replicate samples. In conclusion, for our biological question, we recommend a 50 M read depth in order to obtain a truly comprehensive measure of differential gene expression. However it is important to use biological replicates in order to increase power and ensure results are generalizable to the population level [19,55].

### RNA-seq allows us to profile non-exonic expression

In addition to higher sensitivity and dynamic range, RNA-seq differs from microarrays in its ability to detect expression from areas of the genome regardless of prior annotation, allowing the detection of novel areas of transcription. Such areas, initially dubbed as transcribed “dark matter” [39], are becoming the focus of much attention and debate, fuelled in part by the recent ENCODE project, which showed that a large proportion of the non-exonic region of the human genome was transcribed in at least one cell line [41].

Our read mapping statistics suggest active transcription from non-exonic areas in rat DRGs: only 56-58% of the mappable reads aligned with previously annotated exons, 7-9% of the reads aligning to intronic regions (Figure 2C) and 33-35% aligning outside of the boundaries of annotated genes, i.e. intergenic areas. It would be interesting to perform this experiment in another organism used extensively in pain research, such as mouse, in order to investigate how the proportion of non-exonic reads compare. A possible cause for the high proportion of non-exonic reads may be due to yet unknown tissue-specific gene expression in DRG.

Remarkably, we observed a consistent increase in the proportion of reads mapping to regions of the genome annotated as intronic in SNT samples compared to naive samples, from 7 to 9% of the total number of mapped reads. On a gene-by-gene basis, ten times as many genes showed a significant increase in the proportion of reads mapping to intronic regions in SNT samples compared to naive (Additional file 5). Taken together, these data suggest that the SNT procedure is associated with an increase in expression from intronic regions. Such regions may represent novel exons whose inclusion into mature transcripts is induced upon peripheral nerve injury: the transcriptome of injured DRGs may contain novel exons that have not previously been observed in rat tissues, and are therefore not included in the rat transcriptome annotation.

In our alignment procedure, reads aligning within the genomic coordinates of an annotated gene are ascribed to this gene, however it is also possible they belong to novel genes with overlapping genomic coordinates either in the same or opposite strands (something we cannot determine due to lack of strand information in our protocol). Such novel and nested exons may be protein coding, but also correspond to other RNA species such as microRNAs (miRNAs) or long non-coding RNAs. For example, dysregulation of a number of miRNAs had been demonstrated in experimental models of pain, and a growing body of evidence suggests links between miRNA, spinal nerve injury and pain [10,56-58]. In recent years the study and discovery of long non-coding RNAs has exploded, and at least one such molecule has been shown to have an effect on neuropathic pain [59].

Clearly, these data need further study in order to prioritise potential genes showing SNT-associated non-exonic expression and to confirm a potential role in pain. Prioritisation might include computational methods, such as enrichment studies of the genes showing intronic expression, or sequence based analysis of the intronic RNA, for example looking for potential complementarity to other genomic regions. Such methods would be greatly aided by further, more focused sequencing experiments that use longer, paired end, stranded reads, as well as other techniques to determine the precise start and end site of these RNA species.

Related to the “agnostic” nature of RNA-seq, allowing it to probe unannotated areas of the genome, another useful aspect of RNA-seq data is that it can constantly be reanalysed in light of new genome builds and updated annotations. This would be far harder to achieve for microarray data, because the proportion of the transcribed genome that can be interrogated is constrained by genome annotation at the time of array design: clearly, any genomic area that is not probed in the array chip cannot be measured. Although it would require some modifications to the protocol used in this study, RNA-seq could also be used to compare the transcriptomes of different strains of rat or other organisms, complementing the work of Sorge et al. [60], who compared strains of mice at the genome level and correlated differences in the genome with differences in pain sensitivity.

### Summary of results

We compared RNA-seq and Affymetrix exon array technologies for the purpose of transcriptional profiling of rat DRG tissue after L5 Spinal Nerve Transection. Our key findings were as follows:

1. RNA-seq technology is suitable for the transcriptional profiling of experimental models of pain, as it is able to replicate prior microarray studies.

2. RNA-seq identifies a larger number of DE genes than microarrays, due to its increased sensitivity and higher dynamic range. The number of DE genes identified increases with higher sequencing depth.

3. RNA-seq detects novel areas of transcription mapping to regions not currently annotated as exons (introns and intergenic regions). Some of these regions are differentially expressed in SNT relative to exonic expression and may represent novel candidate pain mediators.

## Conclusions

We have demonstrated that RNA-seq offers major advantages over microarrays for the purpose of whole genome transcriptional profiling of DRG tissue after peripheral nerve injury. Firstly, RNA-seq is more comprehensive as it can interrogate previously unrecognized areas of transcription, while microarray design is constrained to known or predicted transcripts. Secondly, RNA-seq has a much wider dynamic range which favours detection and estimation of differential expression for highly expressed genes and, provided read depth is adequate, lowly expressed genes can also be measured more precisely. Most importantly, RNA-seq provided novel insights into putative novel pain mediators that were not detected using microarrays. We therefore highly recommend the use of RNA-seq for high throughput transcriptional profiling of pain models, and we expect that this technology will supersede microarrays in the near future.

## Methods

### Surgery and tissue collection

Spinal nerve transection (SNT) of the L5 spinal nerve was performed on male Wistar rats (n = 12) as described in [33]. L5 dorsal root ganglia (DRG) were harvested 7 days after surgery by fresh dissection, immediately frozen in liquid nitrogen and stored at -80°C. L5 DRG tissue from naive animals (n = 12) was used as control. Tissue from 4 animals was pooled to create three independent biological replicates per group (SNT or naive) and total RNA was extracted using the miRNEasy kit (QIAGEN, Redwood City, CA, USA) according to manufacturer’s instructions. RNA concentration was measured using the NanoDrop 1000 Spectrophotometer (Thermo Scientific, Wilmington, DE, USA). RNA integrity was assessed using RNA Nano chips in an Agilent 2100 Bionalyzer (Agilent technologies, Santa Clara, CA, USA); RNA integrity numbers (RIN) were between 8.5 and 9.3. Each RNA sample was separated into two technical replicates; one was further processed for microarray analysis and the other for RNA-seq library preparation. Each RNA-seq library was further subdivided into 3 technical replicates, which were sequenced to three distinct read depths as described below.

### Microarray analysis and data processing

Microarray analysis was performed using the Affymetrix GeneChip Rat Exon 1.0 ST Array (Affymetrix, Santa Clara, CA, USA). Sample preparation and hybridization were performed by UCL genomics, following Affymetrix instructions. The resultant CEL files were processed in R using the oligo Bioconductor package [61]. Background correction, normalisation and summarisation were performed using robust multi-array average (RMA), quantile normalisation and median polish respectively [62]. Summarisation was performed at the exon level (where each probeset corresponds to an exon, with some exons being probed by more than one probeset) or the transcript cluster/gene level, where all probesets from different exons belonging to the same gene were summarised to produce a single transcript cluster measurement. Throughout this manuscript “normalised probe intensity” is used to refer to expression at the transcript cluster or exon level, following all three pre-processing steps. Transcript clusters were summarised and annotated using the official Affymetrix .ps and .mps data-files, obtained from the NetAffx Analysis Center (*http://www.affymetrix.com/analysis/index.affx*) through the getNetAffx() function of the oligo package. Presence/Absence calls were determined at the exon level using the detection above background (DABG) method. A default threshold of p = 0.05 for expression above background was used to assign “present” (p < 0.05) and “absent” calls (p > 0.05).

Detection of differentially expressed genes/exons was performed using the limma Bioconductor package [51]. In order to adjust for multiple testing, the false discovery rate (FDR) was derived from the p-values using the method described in [63]. An FDR of 0.1 is accepted when defining significantly differentially expressed genes. Differential expression analysis results for exon arrays, containing fold changes and FDR can be found in Additional file 8. All array data can be found in GEO using accession numbers GSE53764 and GSE53860 for transcript and probe-set level data, respectively. Differential expression data is also available from PainNetworks [64].

### Probeset confidence levels

Rat exon array probesets are annotated with different evidence levels, which indicate the confidence that the probeset truly represents a transcribed genomic sequence, based on the quality of evidence supporting that claim. Probes labelled “core” refer to probesets probing gene transcripts taken from RefSeq and full-length mRNA GenBank records, “extended” probesets are supported by ESTs or partial mRNAs from databases, and “full” probesets are supported by computational predictions. These probeset confidence scores were assigned at the time of array design. Separate gene and exon-level expression matrices were produced, depending on the probe set confidence levels considered, for: 1) core probes only, 2) core and extended probes, and 3) all probes in the chip (core, extended and full, respectively). Core and extended level confidence probes were used when comparing microarrays with RNA-seq. This level of confidence was chosen because it led to the largest number of differentially expressed genes that could be detected with an FDR of 0.1.

### RNA-seq protocol

cDNA libraries were prepared using the TruSeq™ RNA Sample Preparation Kit (Illumina, San Diego, CA, USA), low throughput protocol: 200 ng of total RNA were subjected to poly(A) enrichment using poly(T)-attached magnetic beads. Poly(A)-enriched RNA was subsequently used for reverse transcription and library preparation according to Illumina’s instructions. Sequencing was performed using the Illumina GAIIx sequencer (Illumina). Each library was sequenced at three distinct read depths (M = million reads/sample): ~17 M (average: 16.6 M; range: 14.7 M-17.8 M), ~25 M (average: 25.5 M reads; range: 23.3 M-27.3 M), and ~50 M (average: 50.7 M; range: 42.8 M-53.7 M). All reads were 34 base pairs in length.

### Read alignment

Reads were aligned to the rat genome as summarised in Figure 2. Low quality reads were discarded using the Illumina quality filter, leaving an average 11.9 M (range: 10.7 M- 13.0 M), 19.2 M (17.7 M-20.4 M) and 36.7 M (32.4 M-38.5 M) million reads/sample. Reads were aligned to the reference genome UCSC *Rattus norvegicus* Rn5 (March 2012) using Bowtie [65]. Up to one mismatch was allowed between the reads and the reference genome. Ambiguously mapping reads (i.e. reads that could be mapped to more than one position in the genome) were discarded. One mismatch was chosen because allowing either 0 or 2 or more mismatches reduced the average percentage of uniquely mapping reads per sample.

### Gene expression quantification

Bowtie output files were imported into R using RSamtools [66]. Reads were classified as exonic if they mapped to an annotated exon, intronic if they mapped within the 5’ and 3’ boundaries of a given gene, but outside annotated exons, or intergenic if they aligned outside known 5’ and 3’ boundaries of annotated genes (Figure 2). Gene expression was estimated using the GenomicFeatures package. Gene expression was quantified in one of two ways: considering exonic reads only, or considering intronic and exonic reads (i.e. all reads mapping within the 5’ and 3’ ends of a gene). For genes whose transcripts had alternative start/stop sites, the combination of 5’ and 3’ coordinates that gave maximal coverage, i.e. included all exons of the gene, was selected.

### Comparing relative frequencies of read counts between naive and SNT samples

In order to compare the numbers of reads mapping to the exonic, intronic and intergenic regions, the overdispersed logistic regression model of Williams (1982) was used [67], due to within-group variability being too high to satisfy the assumptions of a simple binomial test.

### Normalisation and differential gene expression in RNA-seq

DESeq [19] was used for normalization of the RNA-seq counts and calculation of differential gene expression. Count data was normalized by estimating effective library size for each sample. As with the microarray analysis, we estimated FDR using the method of Benjamini and Hochberg. An FDR threshold of 0.1 was used to control for false discoveries. Full results for the differential expression analysis for RNA-seq data, including fold changes, p-values and FDR can be found in Additional file 9. RNA-seq alignments (BAM files) can be found in GEO (accession number: GSE53762). Differential expression data is also available from PainNetworks [64].

### Comparison of gene-level expression between platforms

In order to compare microarray gene expression levels to expression measured by RNA-seq, Ensembl (release 69, Rn4) gene annotation was obtained for each microarray transcript-cluster, thus an expression value was obtained for each Ensembl gene probeable using the Affymetrix array. This was plotted against the RNA-seq Reads Per Kilobase per Million mapped reads (RPKM) value for the same Ensembl gene (Ensembl release 74, Rn5). Because the RNA-seq reads were mapped to Rn5, and the microarray annotation was for Rn4, only genes found in both genome builds were plotted. The RPKM is obtained by counting the number of sequenced reads mapping to the exons of a given gene and normalising by the total length of all exons for that gene and the library size [44]. The use of an FDR of 0.1 is somewhat arbitrary (although commonly used). Therefore, in order to justify the use of and FDR of 0.1, we have repeated the comparison of DE genes detected using various FDR thresholds. Additional file 7 shows the overlap in DE genes found by the different platforms at FDRs of 0.05, 0.1, 0.15 and 0.2. We see a similar pattern for all three FDR values – there is a large overlap between platforms, with comparatively few extra genes found by microarrays only, and a larger number of genes found by RNA-seq only.

### Comparison of exon-level expression between platforms

When comparing exon expression between platforms, the normalised probe intensity for each microarray probeset that mapped to an Ensembl exon was compared to the number of reads mapping to that exon, normalised using the RPKM procedure described in [44]. In the case of more than one probeset mapping to the same Ensembl gene, the probeset exhibiting the largest variance in expression across all samples was used for the comparison, as used in [68]. In order to compare expression at the exon level directly between technologies the RNA-seq alignments considered for this particular analysis were made using the Rn4 version of the genome.

### Changes in non-exonic expression following SNT

In order to identify genes for which non-exonic expression changed significantly following SNT, gene expression calculated using exonic expression was compared to gene expression calculated using exonic and intronic expression as described above. Nested genes were not used for this process – any gene that overlapped with any other gene (on either strand) was excluded. In order to look for genes showing a significant increase or decrease in the proportion of reads aligning to intronic gene regions, we used the DEXSeq package [45]. We considered each gene to consist of two units: exons and introns. The package is then employed to look for genes that show a difference in the relative ratios of exonically and intronically aligned reads between SNT and naive samples. Genes were normalised and dispersion estimated using the standard parameters employed by DEXseq. A count based filter was applied before analysis: any gene with less than 200 reads aligning to its intronic regions for more than 3 samples was excluded from the analysis.

### Functional analysis

For functional enrichment analysis the exon array and the RNA-seq (read depth 50 M) datasets were considered separately. Lists of dysregulated genes (FDR p < 0.1) were subjected to gene ontology analysis. Analysis by “Protein Class” was performed with “PANTHER classification systems” (http://www.pantherdb.org/) [69]. Functional analysis was performed with Ingenuity Pathway Analysis (IPA, QIAGEN). Lists of dysregulated genes were subjected to “core analysis” using IPA default settings, and top biological functions/ “diseases and disorders” as well as “canonical pathways”; p-values were adjusted using the Benjamini-Hochberg multiple testing correction.

### Data access

Raw and processed data are available from the Gene Expression Omnibus (GEO) (http://www.ncbi.nlm.nih.gov/geo/), series accession number: GSE53861. Tables of gene expression and the lists of the DE genes are available from http://www.PainNetworks.org[64] from the experiments tab.

## Abbreviations

DABG: Detection above background; DE: Differentially expressed; FC: Fold change; DRG: Dorsal root ganglia; FDR: False discovery rate; RNA-seq: RNA-Sequencing; RPKM: Reads per kilobase per million mapped reads; SNT: L5 spinal nerve transection.

## Competing interests

The authors declare that they have no competing interests.

## Authors’ contributions

JRP, MK and AAM performed data analysis. AAM performed RNA sample preparation. MC and JG performed SNT surgery. WR and RS performed RNA-seq. DLHB, SBM and CO conceived of and managed this study. All authors read and approved the final manuscript.

## Supplementary Material

Additional file 1 **Inter-platform correlation at the gene level.** Each pdf file in this zipped folder contains plots of RNA-seq RPKM expression vs. microarray normalised probe intensity for all respective samples. Spearman’s correlation coefficient is indicated in the top left corner of each graph. Click here for file

Additional file 2 **The distributions of absolute log**_**2**_** FCs for DE genes, shown alongside the absolute log**_**2**_** FCs for non-DE genes.** Absolute log 2 FCs are shown for the genes that are called as DE by both platforms (red lines, dashed and solid lines show RNA-seq and microarray fold changes), by RNA-seq only (dashed green line) and by microarrays only (solid blue line). Non DE genes shown in grey (dashed line shows RNA-seq values, solid line represents microarray values). Distribution curve computed using the probability density function, implemented in R. Click here for file

Additional file 3 **Comparison of RNA-seq and microarrays for the measurement of exon expression and the detection of differentially expressed exons.** A) Correlation between normalised hybridisation intensity and normalized read counts (RPKM) at a 50 M read depth for exons measureable using microarrays and RNA-seq. Where more than one probeset maps to a given exon, both values are plotted, as separate points, for the equivalent RNA-seq value for that exon. Ai) Average expression for all three SNT samples. Aii) Average expression for all three naive samples. The red points show exons expressed in both platforms, blue points show exons that are not detected by RNA-seq (i.e. 0 reads aligned to that exon). Green points show exons with microarray normalised probe intensity below that of the background probesets (calculated using the DABG measure described in the Methods section), but with an RNA-seq RPKM value above 0. Grey points show exons with microarray normalised probe intensity below that of background probesets, and with an RPKM of 0. Some noise has been added to the expression values of the exons for clearer visualization of the point density. B) Correlation between fold changes estimated by microarrays and RNA-seq (50 M read depth) for exons detectable by both technologies. Exons deemed as significantly DE by both platforms are shown as red points; exons detected as DE exclusively by RNA-Seq are shown as green points; exons detected as DE exclusively by microarrays are shown as blue points. C) Venn diagram showing the number of exons found to be differentially expressed by RNA-seq (shown for a read depth of 50 M) and the overlap with microarray data. Click here for file

Additional file 4 **Overlap between platforms at the exon level, for all sequencing depths.** Number of exons called as DE for RNA-seq, microarrays and the overlap between them. For lower sequencing depths, microarrays call more exons as DE. Click here for file

Additional file 5 **Fold change at exonic and intronic levels and p-values.** Table containing fold changes calculated by DEXseq at intronic and exonic level and DEXSeq p-values for all genes tested. Click here for file

Additional file 6 **Volcano and mean-fold change plot for the DEXSeq-based analysis of relative exonic vs. intronic expression.** A) Volcano plot, which shows the logarithm of the change in exonic expression minus the change in intronic expression, following SNT (x-axis). This is plotted against the negative logarithm of the p-value (y-axis). We can see that the most significant genes (i.e. those with the highest value on the y-axis) are represented by points with a negative value on the x-axis; this suggests that the most affected genes in terms of intronic vs. exonic expression are showing an increased expression in intronic regionsfollowing SNT. B) Plot of mean intronic expression vs. the logarithm of the change in exonic expression minus the change in intronic expression, highlighting the genes that have been deemed significant (FDR < 0.1), showing that significance is not a function of expression. Click here for file

Additional file 7 **The effect of changing the permitted false discovery rate, on the total number of genes deemed as differentially expressed.** Numbers of genes called as significantly DE for RNA-seq, microarrays and the overlap between them for varying FDRs. Ensembl gene ids and gene symbols are given. Click here for file

Additional file 8 **Exon array limma analysis, containing the Ensembl gene ids and gene symbols (obtained from NetAffx and Biomart).** Results produced using the limma package. In the case of more than one transcript cluster id with the same Ensembl id, the transcript cluster showing the highest level of variation across samples was used for the limma analysis. Results are shown for extended level confidence probes. Click here for file

Additional file 9 **RNA-seq (50 M) DESeq results containing the Ensembl gene ids and gene symbols (obtained from NetAffx and Biomart).** Results produced using the DESeq package, using the default normalization parameters. Genes to which no reads could be aligned for four or more samples were excluded from analysis. Click here for file
