# Supplementary material for: A comparison of RNA-seq and exon arrays for whole genome transcription profiling of the L5 spinal nerve transection model of neuropathic pain in the rat
Source: Mol Pain. 2014 Jan 28;10:7. doi: 10.1186/1744-8069-10-7 (PMC4021616; doi:10.1186/1744-8069-10-7)
Supplement: Additional file 1 — Inter-platform correlation at the gene level. Each pdf file in this zipped folder contains plots of RNA-seq RPKM expression vs. microarray normalised probe intensity for all respective samples. Spearman’s correlation coefficient is indicated in the top left corner of each graph. [file 1744-8069-10-7-S1.zip › Additional File 1/InterPlatformGeneLevel_25Mextd.pdf]

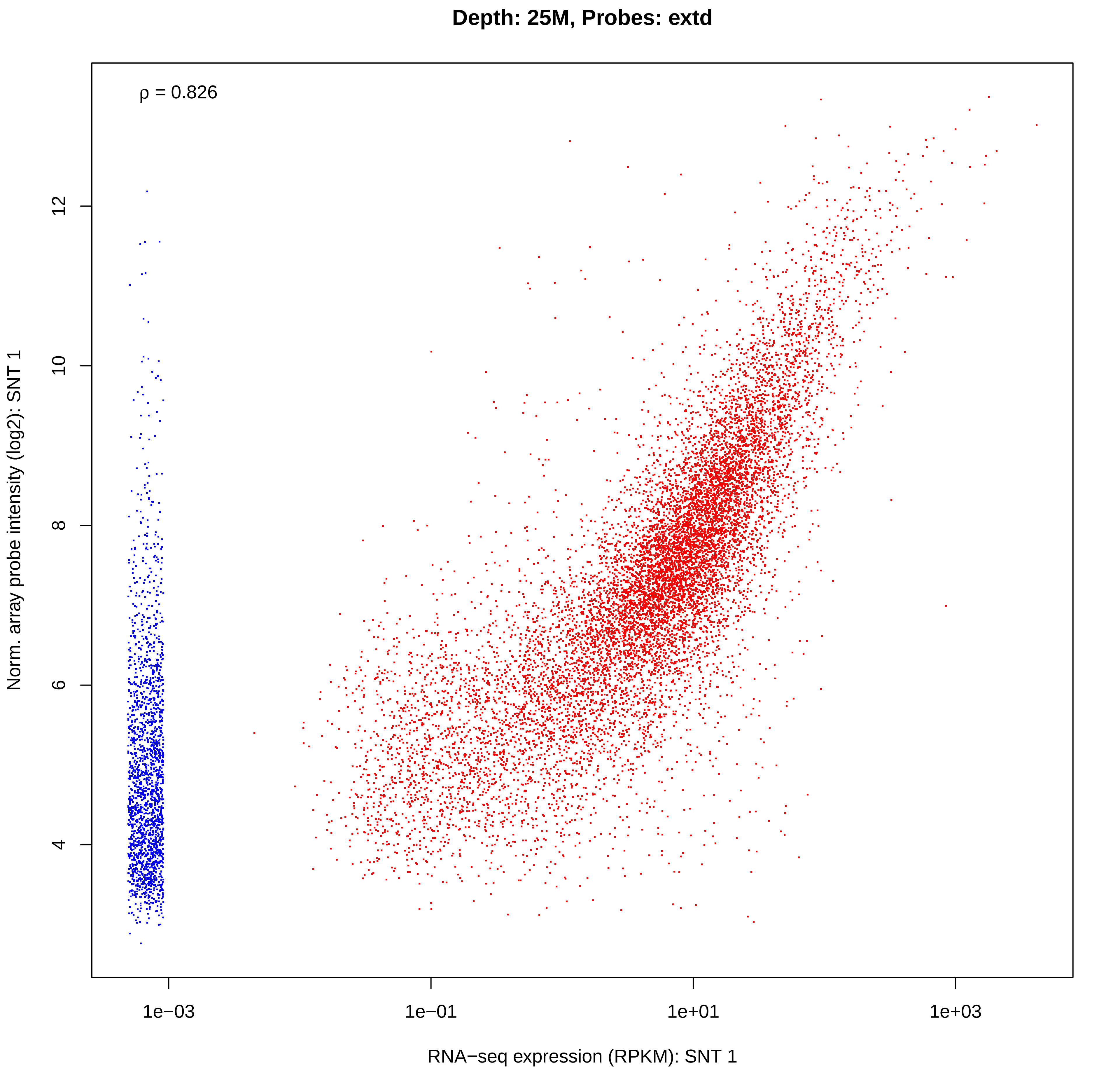
Depth: 25M, Probes: extd
ρ = 0.826
Norm. array probe intensity (log2): SNT 1
4
6
8
10
12
1e−03
1e−01
1e+01
1e+03
RNA−seq expression (RPKM): SNT 1

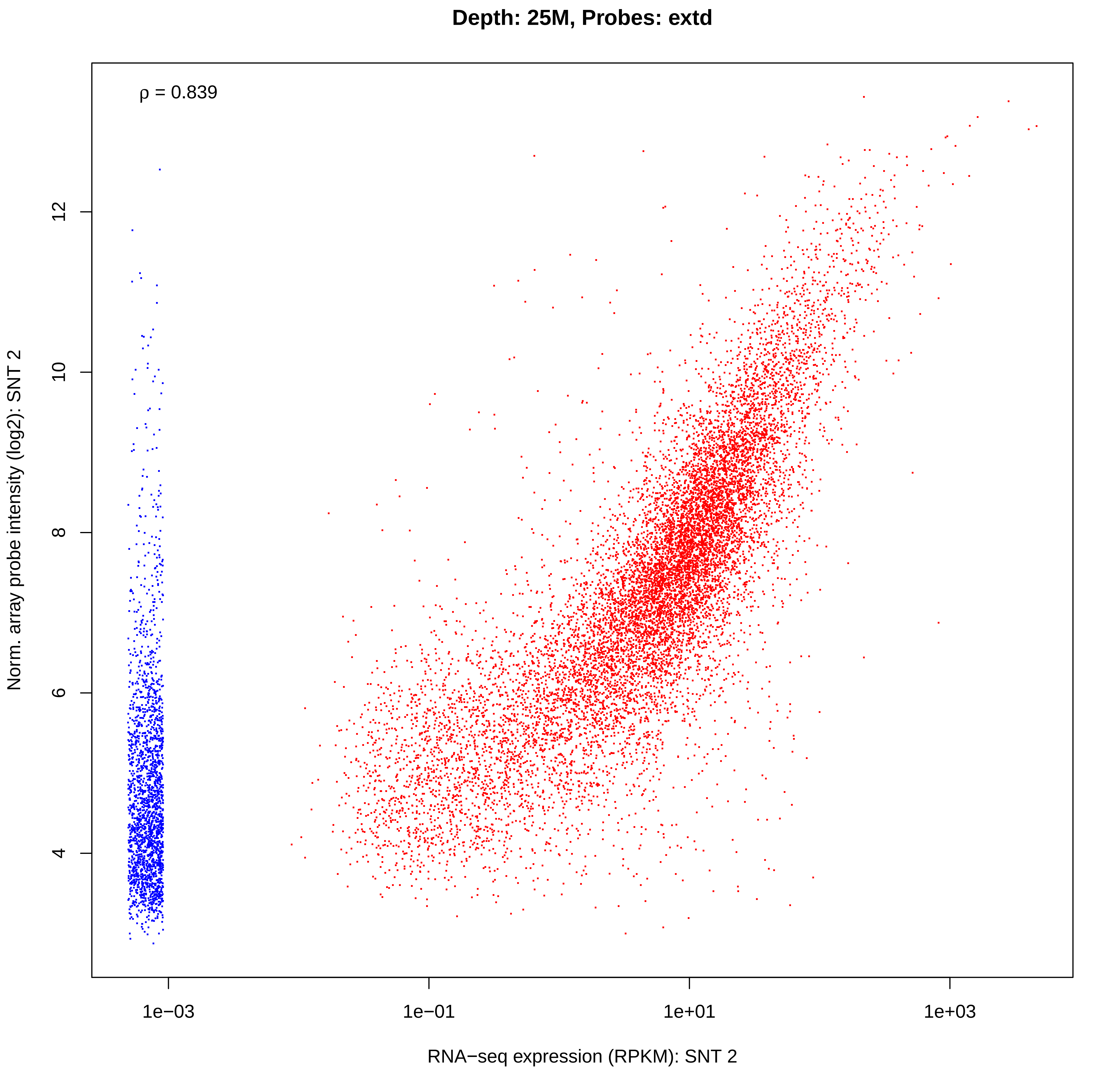

Depth: 25M, Probes: extd
ρ = 0.839
Norm. array probe intensity (log2): SNT 2
4
6
8
10
12
RNA−seq expression (RPKM): SNT 2
1e−03
1e−01
1e+01
1e+03

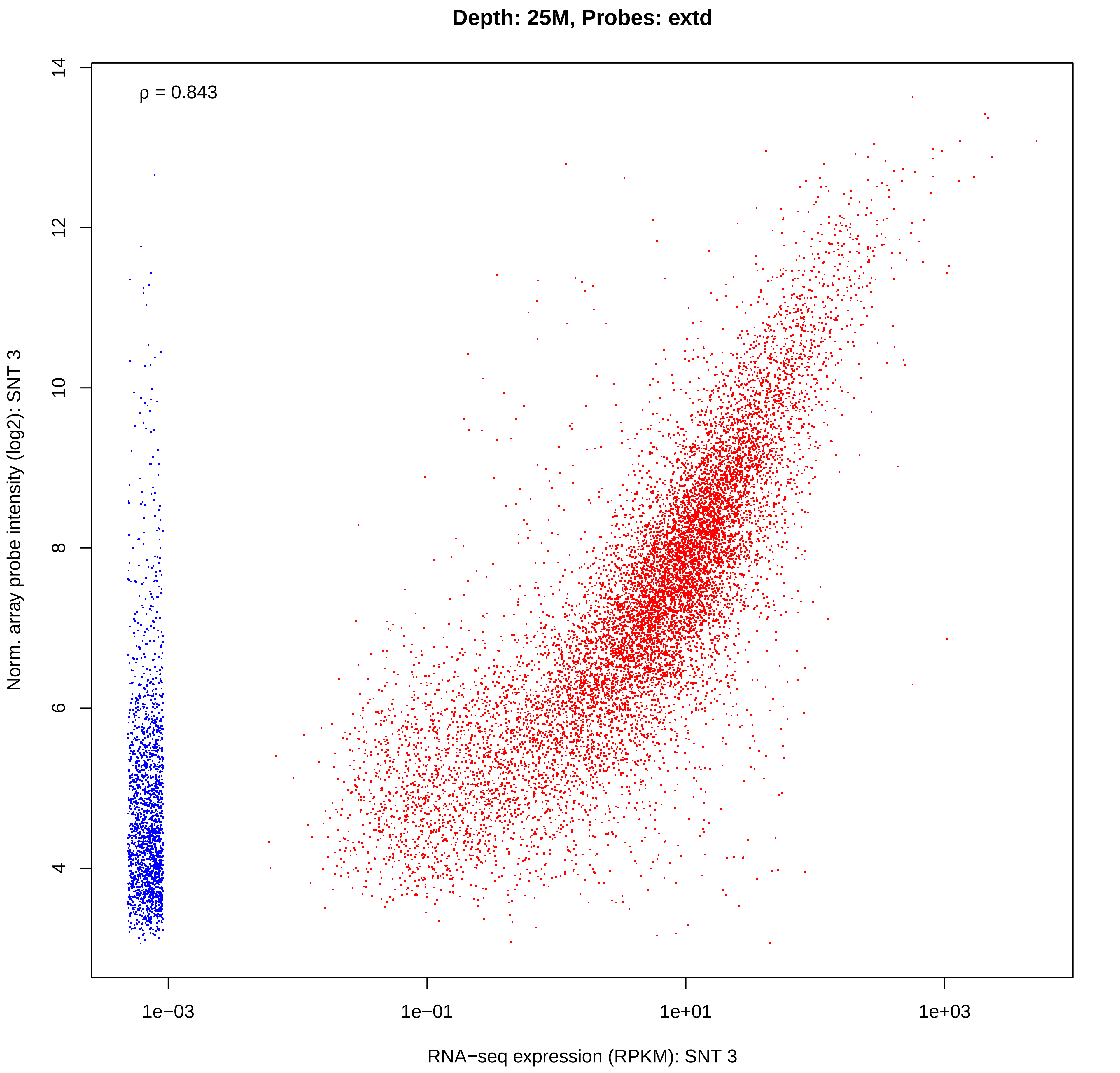

Depth: 25M, Probes: extd
ρ = 0.843
Norm. array probe intensity (log2): SNT 3
4
6
8
10
12
14
1e−03
1e−01
1e+01
1e+03
RNA−seq expression (RPKM): SNT 3

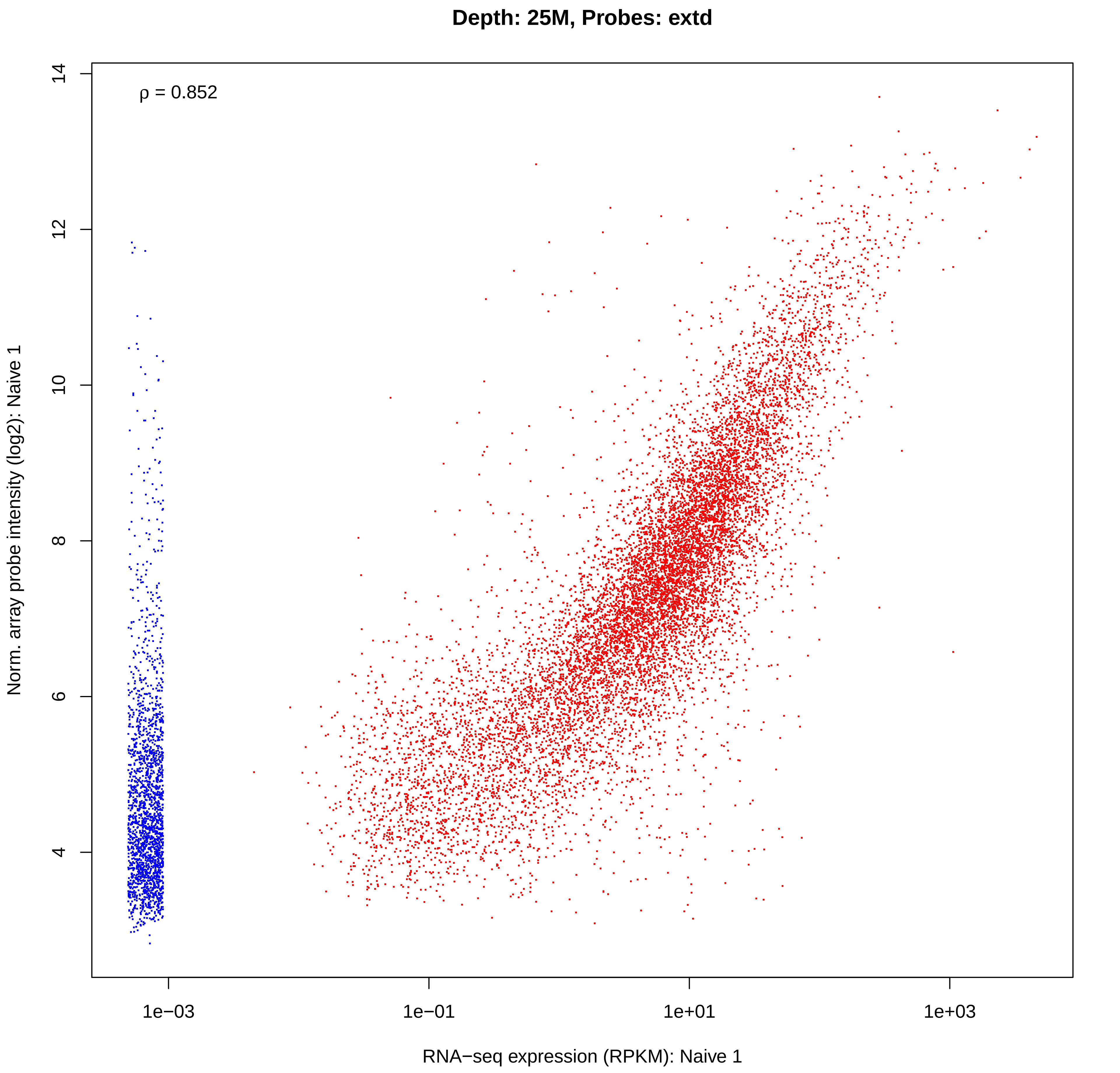
Depth: 25M, Probes: extd
ρ = 0.852
Norm. array probe intensity (log2): Naive 1
4
6
8
10
12
14
1e−03
1e−01
1e+01
1e+03
RNA−seq expression (RPKM): Naive 1

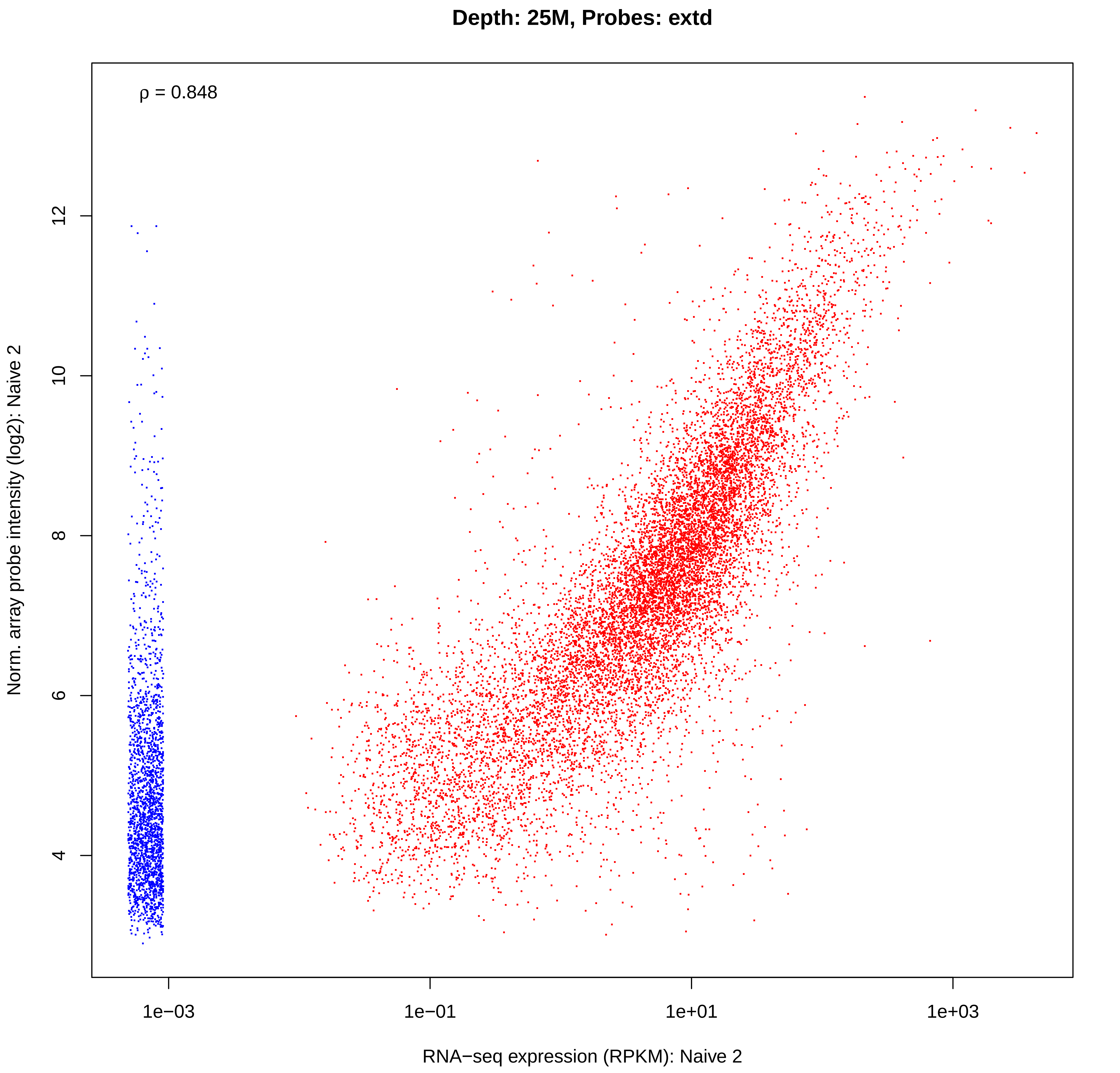
Depth: 25M, Probes: extd
ρ = 0.848
Norm. array probe intensity (log2): Naive 2
4
6
8
10
12
1e−03
1e−01
1e+01
1e+03
RNA−seq expression (RPKM): Naive 2

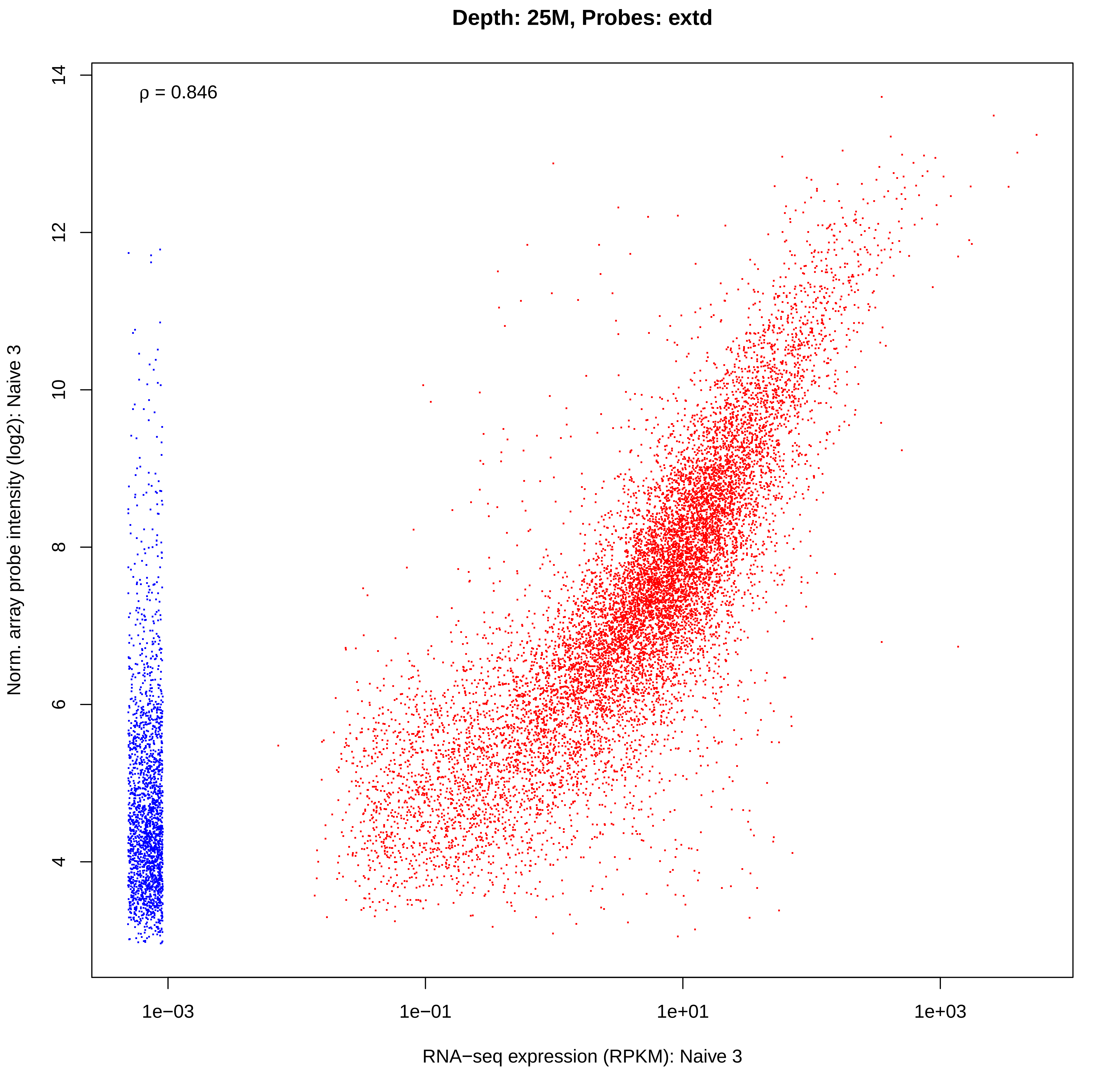
Depth: 25M, Probes: extd
ρ = 0.846
Norm. array probe intensity (log2): Naive 3
14
12
10
8
6
4
1e−03
1e−01
1e+01
1e+03
RNA−seq expression (RPKM): Naive 3
