# Supplementary figures and images for: A comparison of RNA-seq and exon arrays for whole genome transcription profiling of the L5 spinal nerve transection model of neuropathic pain in the rat
Source: Mol Pain. 2014 Jan 28;10:7. doi: 10.1186/1744-8069-10-7 (PMC4021616; doi:10.1186/1744-8069-10-7)

Density

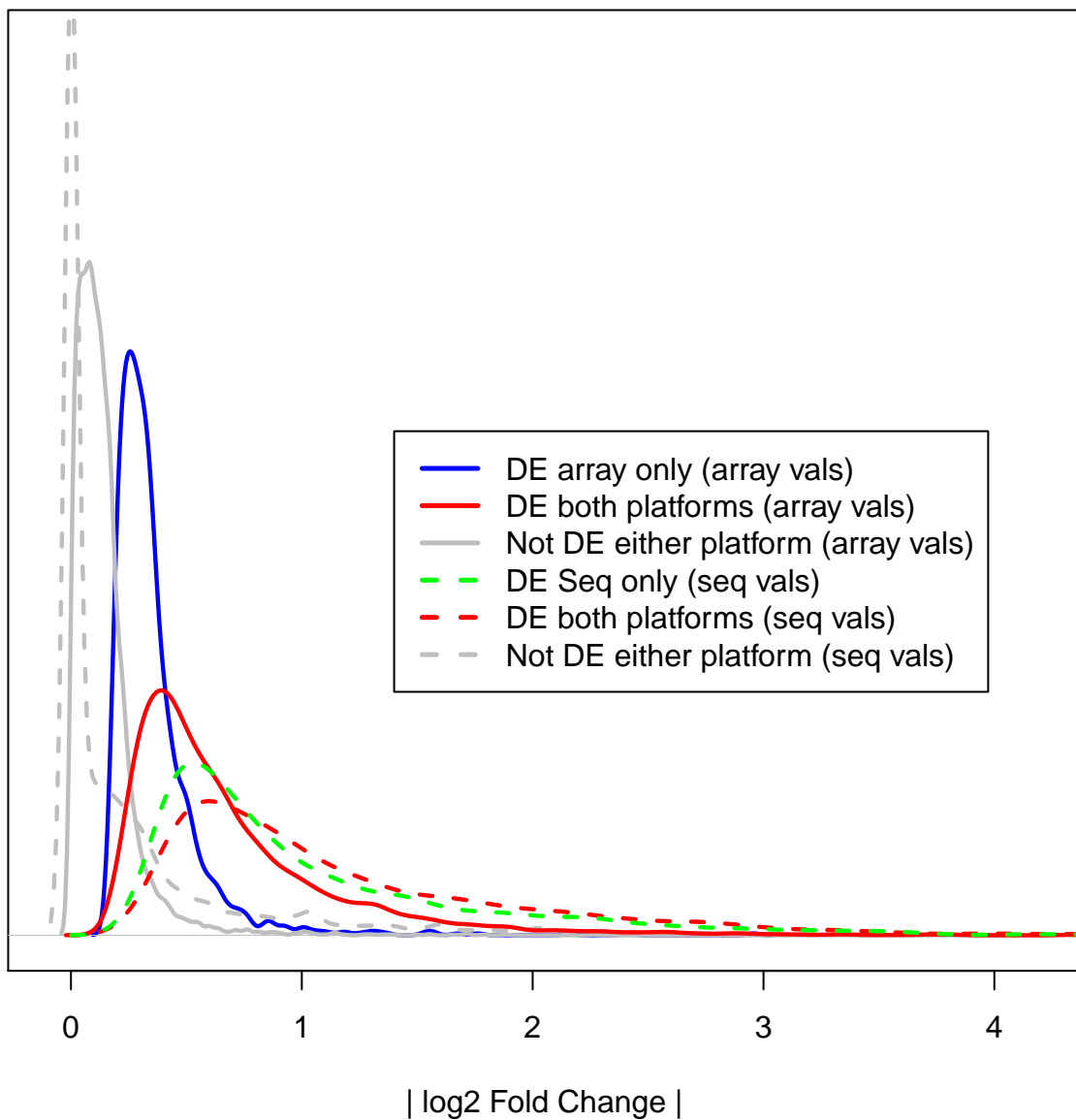

Supplement: Additional file 2 — The distributions of absolute log2 FCs for DE genes, shown alongside the absolute log2 FCs for non-DE genes. Absolute log 2 FCs are shown for the genes that are called as DE by both platforms (red lines, dashed and solid lines show RNA-seq and microarray fold changes), by RNA-seq only (dashed green line) and by microarrays only (solid blue line). Non DE genes shown in grey (dashed line shows RNA-seq values, solid line represents microarray values). Distribution curve computed using the probability density function, implemented in R. [file 1744-8069-10-7-S2.pdf]

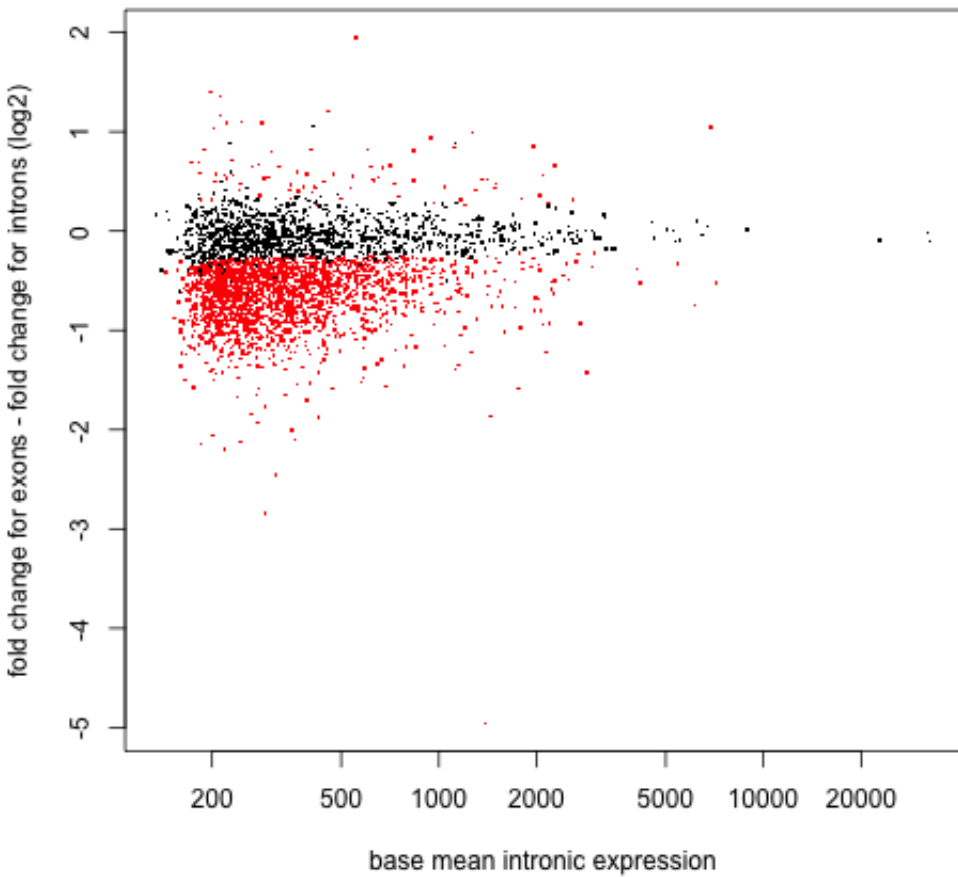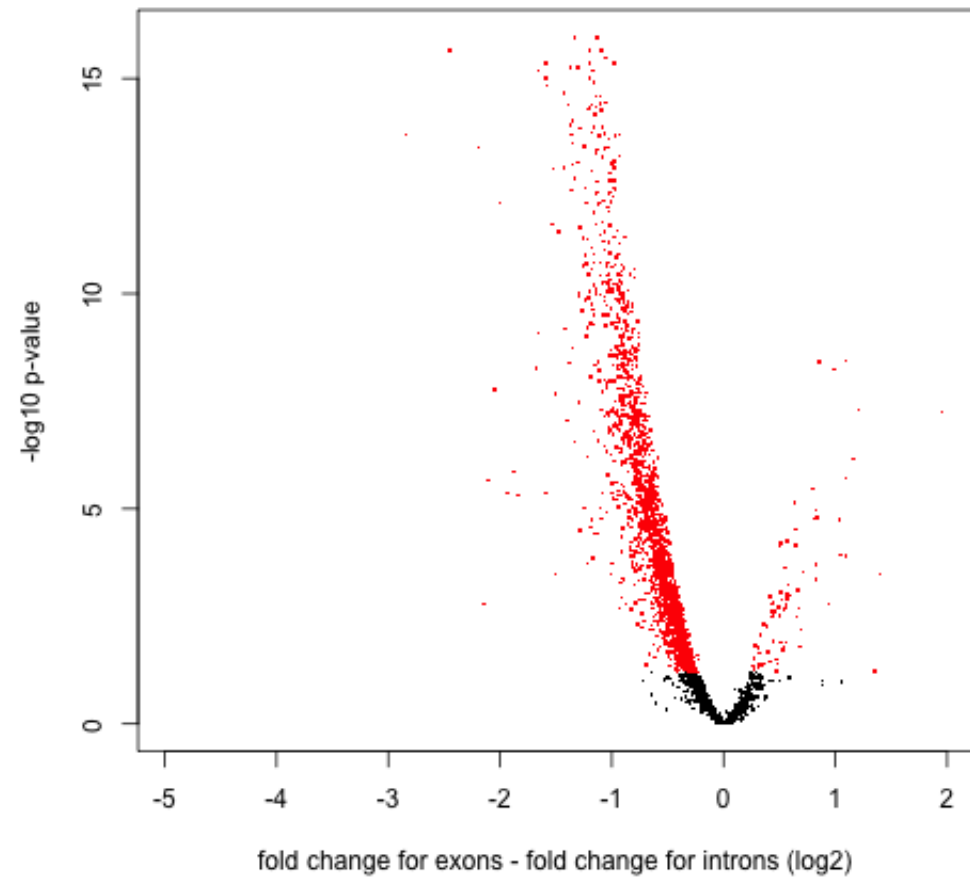

Supplement: Additional file 6 — Volcano and mean-fold change plot for the DEXSeq-based analysis of relative exonic vs. intronic expression. A) Volcano plot, which shows the logarithm of the change in exonic expression minus the change in intronic expression, following SNT (x-axis). This is plotted against the negative logarithm of the p-value (y-axis). We can see that the most significant genes (i.e. those with the highest value on the y-axis) are represented by points with a negative value on the x-axis; this suggests that the most affected genes in terms of intronic vs. exonic expression are showing an increased expression in intronic regionsfollowing SNT. B) Plot of mean intronic expression vs. the logarithm of the change in exonic expression minus the change in intronic expression, highlighting the genes that have been deemed significant (FDR < 0.1), showing that significance is not a function of expression. [file 1744-8069-10-7-S6.pdf]
