# Supplementary material for: A comparison of RNA-seq and exon arrays for whole genome transcription profiling of the L5 spinal nerve transection model of neuropathic pain in the rat
Source: Mol Pain. 2014 Jan 28;10:7. doi: 10.1186/1744-8069-10-7 (PMC4021616; doi:10.1186/1744-8069-10-7)
Supplement: Additional file 3 — Comparison of RNA-seq and microarrays for the measurement of exon expression and the detection of differentially expressed exons. A) Correlation between normalised hybridisation intensity and normalized read counts (RPKM) at a 50 M read depth for exons measureable using microarrays and RNA-seq. Where more than one probeset maps to a given exon, both values are plotted, as separate points, for the equivalent RNA-seq value for that exon. Ai) Average expression for all three SNT samples. Aii) Average expression for all three naive samples. The red points show exons expressed in both platforms, blue points show exons that are not detected by RNA-seq (i.e. 0 reads aligned to that exon). Green points show exons with microarray normalised probe intensity below that of the background probesets (calculated using the DABG measure described in the Methods section), but with an RNA-seq RPKM value above 0. Grey points show exons with microarray normalised probe intensity below that of background probesets, and with an RPKM of 0. Some noise has been added to the expression values of the exons for clearer visualization of the point density. B) Correlation between fold changes estimated by microarrays and RNA-seq (50 M read depth) for exons detectable by both technologies. Exons deemed as significantly DE by both platforms are shown as red points; exons detected as DE exclusively by RNA-Seq are shown as green points; exons detected as DE exclusively by microarrays are shown as blue points. C) Venn diagram showing the number of exons found to be differentially expressed by RNA-seq (shown for a read depth of 50 M) and the overlap with microarray data. [file 1744-8069-10-7-S3.pdf]

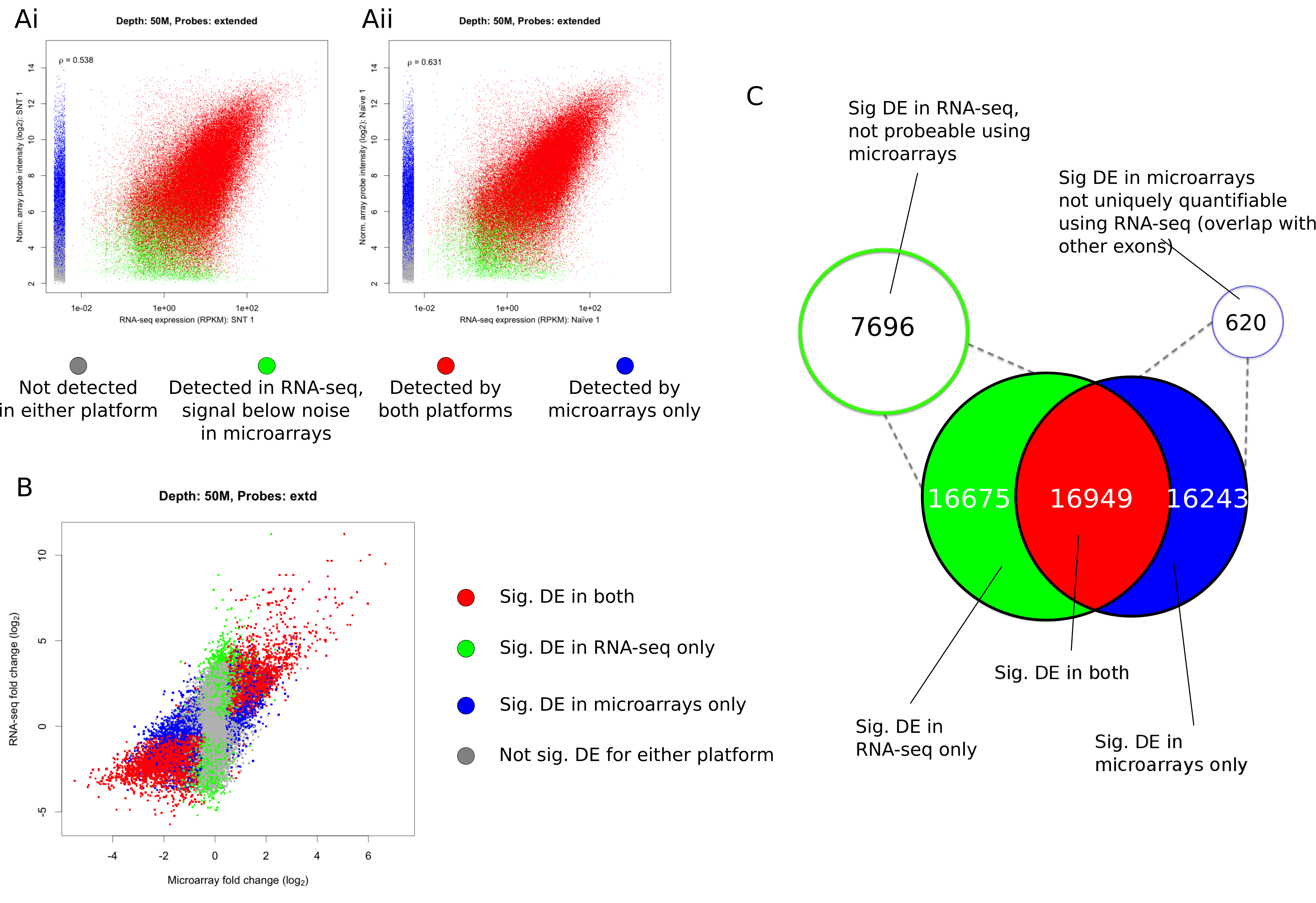
Ai
Depth: 50M, Probes: extended
ρ = 0.538
Norm. array probe intensity (log2): SNT 1
RNA-seq expression (RPKM): SNT 1
Aii
Depth: 50M, Probes: extended
ρ = 0.631
Norm. array probe intensity (log2): Naïve 1
RNA-seq expression (RPKM): Naïve 1
Not detected in either platform
Detected in RNA-seq, signal below noise in microarrays
Detected by both platforms
Detected by microarrays only
B
Depth: 50M, Probes: extd
RNA-seq fold change (log2)
Microarray fold change (log2)
Sig. DE in both
Sig. DE in RNA-seq only
Sig. DE in microarrays only
Not sig. DE for either platform
C
Sig DE in RNA-seq, not probeable using microarrays
7696
Sig DE in microarrays not uniquely quantifiable using RNA-seq (overlap with other exons)
620
16675
16949
16243
Sig. DE in both
Sig. DE in RNA-seq only
Sig. DE in microarrays only
